# Integrated sequence and -omic features reveal novel small proteome of *Mycobacterium tuberculosis*

**DOI:** 10.3389/fmicb.2024.1335310

**Published:** 2024-05-15

**Authors:** Priyansha Raj Sinha, Rami Balasubramanian, Shubhada R. Hegde

**Affiliations:** Institute of Bioinformatics and Applied Biotechnology (IBAB), Bengaluru, India

**Keywords:** small proteins, RNA-Seq, Ribo-Seq, coupled ORFs, Random Forest

## Abstract

Bioinformatic studies on small proteins are under-represented due to difficulties in annotation posed by their small size. However, recent discoveries emphasize the functional significance of small proteins in cellular processes including cell signaling, metabolism, and adaptation to stress. In this study, we utilized a Random Forest classifier trained on sequence features, RNA-Seq, and Ribo-Seq data to uncover small proteins (smORFs) in *M. tuberculosis*. Independent predictions for the exponential and starvation conditions resulted in 695 potential smORFs. We examined the functional implications of these smORFs using homology searches, LC-MS/MS, and ChIP-seq data, testing their expression in diverse growth conditions, and identifying protein domains. We provide evidence that some of these smORFs could be part of operons, or exist as upstream ORFs. This expanded data resource for the proteins of *M. tuberculosis* would aid in fine-tuning the existing protein and gene regulatory networks, thereby improving system-wide studies. The primary goal of this study was to uncover and characterize smORFs in *M. tuberculosis* through bioinformatic analysis, shedding light on their functional roles and genomic organization. Further investigation of these potential smORFs would provide valuable insights into the genome organization and functional diversity of the *M. tuberculosis* proteome.

## Introduction

Small proteins of length 5–100 amino acids are encoded by small open reading frames (ORFs), often independent of the downstream ORF. Some known small proteins include ribosomal proteins, nucleic acid chaperones, and kinase regulators. Many small proteins are essential for cellular activities such as cell division, signal transmission, transportation, and sporulation (Storz et al., 2014). Additional functions of the small proteins include functions such as toxins, being part of larger protein complexes, interacting with the membrane, controlling the activities of transcription regulators, and binding to DNA or RNA. The evolutionary characterization of small proteins reveals intriguing trends. Small proteins tend to be more evolutionarily dynamic than longer proteins, showing elevated Ka/Ks ratios in interspecific comparisons and suggesting rapid evolution. While some small proteins evolve into compact multi-domain proteins, others remain small, suggesting diverse evolutionary pathways (Su et al., 2013). Their small size and versatility make them well-suited for such diverse roles (Gray et al., 2022). For instance, the tarsal-less (*tal*) gene in *Drosophila* encodes an 11-amino acid peptide that controls gene expression and tissue folding (Cheng et al., 2011). Some small proteins interact with other proteins to modify their structural conformation, which subsequently govern their stabilities and enzymatic activities. For example, Sda (46 amino acids) in *Bacillus subtilis* interacts with histidine kinase KinA and induces a change in its conformation, which results in the inhibition of KinA autophosphorylation (Rowland et al., 2004; Whitten et al., 2007; Storz et al., 2014). Similarly, conserved small proteins SpoVM and CmpA exist in endospore-forming bacteria and exert significant influence on sporulation processes. In *Escherichia coli*, SpoVM obstructs the function of FtsH, an essential factor for initiating sporulation (Cutting et al., 1997). Also, a PhoQP-regulated small protein MgrB in *E. coli* negatively regulates PhoQ sensor kinase (Lippa and Goulian, 2009). Moreover, CmpA from *B. subtilis* impacts coat and cortex morphogenesis during sporulation. Deleting *cmpA* speeds up sporulation, while overproduction delays the entry into the sporulation stage and interferes with cortex assembly (Ebmeier et al., 2012). Other *B. subtilis* small proteins MciZ and FsrA regulate cell division and iron homeostasis, respectively (Handler et al., 2008; Smaldone et al., 2012). In another study, two previously unidentified low-mass proteins, TB7.3 and TB10.4, were discovered, with genetic analysis confirming that TB10.4, in conjunction with the previously recognized small protein CFP10, belongs to the low-mass ESAT-6 family, while TB7.3 stands apart as a low-molecular-mass protein outside of this family (Skjøt et al., 2000).

Some regulatory small RNAs (sRNAs) that also encode small proteins are termed dual-function sRNAs. These sRNAs can influence target gene expression through various mechanisms, including antisense activity via complementary base-pairing, modulation of RNA binding protein activities, and translation into peptides that participate in either similar or distinct metabolic processes as the target gene (Thomason and Storz, 2010; Raina et al., 2018). One such dual-function sRNA is SgrS in *E. coli*, which prevents the synthesis of glucose transporters and indirectly promotes the translation of sugar phosphatase. The sRNA also codes for a protein SgrT, which inactivates existing glucose transporters through protein-protein interactions (Wadler and Vanderpool, 2007). Another example is SR1 in *B. subtilis*, which initially known for inhibiting translation initiation of *ahrC* mRNA, was later found to encode SR1P, a highly conserved small peptide. SR1P binds to GapA, stabilizing the *gapA* operon mRNA rather than promoting translation, thus showcasing a unique dual functionality in *B. subtilis* (Gimpel et al., 2010). Additionally, the McaS sRNA of *E. coli* demonstrates dual functionality by directly base-pairing with mRNA targets and indirectly regulating gene expression by binding to the global regulator CsrA. This unique role in controlling biofilm formation adds complexity to the post-transcriptional regulatory network (Jørgensen et al., 2013). Interest in identifying and characterizing small proteins has increased recently due to their functional importance.

Previously, comparative genomics approaches were employed to identify small proteins in a given genome (Hemm et al., 2008; Cheng et al., 2011; Samayoa et al., 2011; Yang et al., 2011). One notable tool RanSEP utilizes the Random Forest method to identify potential small proteins in bacterial genomes based on sequence-level coding features (Miravet-Verde et al., 2019). Additionally, conserved small ORFs were identified through studies combining comparative genomics, sequence features, and ribosome profiling data (Crappé et al., 2013; Friedman et al., 2017). The advent of sequencing technology has facilitated the comprehensive identification and characterization of numerous small proteins. For instance, ribosome profiling data has revealed signatures of translation for many small ORFs (Ingolia, 2014). To annotate prokaryotic genomes, DeepRibo utilizes ribosome profiling as a means of predicting expressed protein-coding genes (Clauwaert et al., 2019). FSPP is another high-throughput small ORF predictor that employs mass spectrometry data, RNA-Seq, and Ribo-Seq data to predict genome-wide small proteins in humans (Li et al., 2018). These existing tools primarily rely on sequence features or expression data from Ribo-Seq or RNA-Seq.

There are a couple of studies focused on genome-wide small protein identification in a given bacterial genome. For instance, in *E. coli*, intergenic small proteins were identified through a combination of conservation analysis and ribosome binding site models (Hemm et al., 2008; VanOrsdel et al., 2018). Furthermore, ribosome profiling data enabled the genome-wide identification of small ORFs in *E. coli* (Weaver et al., 2019). In *M. smegmatis*, small proteins encoded by upstream open reading frames (uORFs) were identified, suggesting their potential role in enhancing the translation initiation of the downstream genes (Shell et al., 2015). Additionally, Ribo-Seq data analysis in *M. abscessus* identified about 113 small ORFs, the majority of which show conservation across other mycobacterial species (Miranda-CasoLuengo et al., 2016). In mycobacteria, a few small proteins are differentially expressed across growth conditions. For example, MAB_5038c and MAB_5039c of *M. abscessus* are upregulated in the artificial sputum and upon exposure to erythromycin or kanamycin, and not in hypoxia (Miranda-CasoLuengo et al., 2016). Many studies describe the role of small proteins in *M. tuberculosis*. ESX proteins, crucial virulence factors in *M. tuberculosis*, facilitate protein secretion and host-pathogen interactions. EsxA, a small protein co-expressed with the ESX-1 secretion system, is vital for *M. tuberculosis* virulence, playing a role in substrate secretion and ESX-1 gene expression regulation (Bao et al., 2021). Also, *M. tuberculosis* small heat shock proteins Arc1 (also known as Hsp16.3/16-kDa antigen and α-crystallin-related protein 1) and Arc2 (also called HrpA) are highly expressed during the non-replicative phase and upon heat shock stress, respectively (Kennaway et al., 2005). Therefore, conditional expression of these small proteins suggests their role in the host adaptation of mycobacteria.

Despite all these approaches, a comprehensive, organism-specific, genome-wide identification of small proteins at higher confidence remains a challenge. We developed a machine-learning pipeline by integrating genomic features with the gene expression and ribosome binding information in a single framework to identify small proteins in *M. tuberculosis*. This comprehensive approach revealed 695 small proteins (smORFs henceforth), many of which are conserved in other bacterial species. We reinforced many of their functional importance by analyzing proteomics data, predicting the conserved domains, and deriving functional annotations. By comparing our results with previous findings, we highlight the novelty of our approach. We compared our predictions with those of previous tools, demonstrating improved performance. Furthermore, we predict small proteins under two conditions, exponential and starvation, offering insights into potential therapeutic targets and enhancing our understanding of *M. tuberculosis* pathogenicity.

## Methods

### Feature selection for Random Forest classification

Combined sequence features, expression (RNA-Seq), and ribosome binding (Ribo-Seq) signatures were used to predict small proteins in *M. tuberculosis*. Independent predictions were made for the exponential and starvation growth conditions using corresponding RNA-Seq and Ribo-Seq data while keeping the sequence features constant. *M. tuberculosis* RNA-seq and Ribo-seq data at exponential growth and 24 h of nutrient starvation were downloaded from the European Nucleotide Archive (ENA) (https://www.ebi.ac.uk/ena) (Sawyer et al., 2021) (E-MTAB-8835). Using Trimmomatic (v0.39), adapters from the raw reads were trimmed and the reads were aligned against *M. tuberculosis* H37Rv reference genome (NC_000962.3) using Bowtie2 (v2.3.4.1) (Langmead and Salzberg, 2012; Bolger et al., 2014). Aligned SAM files were sorted, indexed, strand separated, and converted into BAM files, and the replicates were merged using samtools (v1.7) (Li et al., 2009). Expression of the ORFs was calculated as normalized RPKM from the read counts obtained using MultiBamCov in bedtools (v2.26.0) (Quinlan and Hall, 2010). Likewise, Ribosome binding of the ORFs was assessed using the same approach. Protein-coding sequence features were extracted from the Python package iFeature (Chen et al., 2018). All the sequence feature outputs provided by the iFeature Python package were normalized values. Of the 18 different features provided by iFeature, eight best features that efficiently separate positive and negative training sets were chosen. These include hydrophobicity, normalized Van der Waals volume, polarity, polarizability, charge, grouped amino acid composition (GAAC), secondary structures, and solvent accessibility. To this list, GC content was included as an additional sequence-based feature. The features on which the classifier is trained are not specific to small proteins, but test the coding potential of the genomic regions with start and stop codons in-frame.

For training the machine learning model, 197 and 193 annotated proteins of *M. tuberculosis* with amino acid length ≤ 100 which are expressed and ribosome-bound in exponential and stationary growth phases, respectively, were considered as a positive set. Proteins were recognized as expressed and ribosome-bound if their expression and ribosome binding (measured as RPKM) values surpassed the median value of all the annotated proteins in *M. tuberculosis*. The negative training set was comprised of 200 random *in silico* translated sequences generated from the stop codon to the start codon located between the annotated ORFs in *M. tuberculosis*. For generating test data, *M. tuberculosis* ORFeome comprising all possible ORFs in the genome with a length cut-off of ≥ 30 bases was generated using codon translation table 11, with start codons ATG, TTG, and GTG and stop codons TAG, TAA, and TGA. The test set contained ~85% potential ORFs with a size of <100 amino acids, indicating a bias toward smaller proteins due to the absence of a length filter. A total of 74,976 potential ORFs along the genome spanning both the strands were generated. Novel ORFs encoding small proteins were predicted from 45,652 potential ORFs (test set) devoid of annotated protein-coding regions, repeat elements, ORFs with > 75% overlap with the annotated protein-coding regions, and tRNA and rRNA-coding regions. Random Forest classifier method (Ho, 1995) of the python package scikit-learn (0.21.2) (Pedregosa et al., 2011) was used for the prediction. The classifier performance in cross-validation was calculated using the cross_val_score function in the scikit-learn library. The mean values of the cross-validation score in a 5-fold CV in the exponential and stationary growth phase data were 0.98 and 0.99, respectively. Hyperparameter tuning was performed to decide the best parameters for the prediction which included n_estimators=1000 and max_depth=10. Accuracy was derived based on the AUC of the ROC curves. A probability cutoff of 0.6 was used to list the final predictions.

### Comparison with the previously reported methods

The ORFs predicted in *M. tuberculosis* by the previously reported tools RanSEPs (Miravet-Verde et al., 2019), DeepRibo (Clauwaert et al., 2019), and a ribosome profiling study conducted by Smith et al. (2022) were obtained. RanSEPs uses sequence features to detect small proteins in a given bacterial genome. With a probability score (RanSEPs score) cutoff of > 0.6, RanSEPs predicted 6494 smORFs in *M. tuberculosis* which are devoid of the annotated proteins. DeepRibo is a neural network-based tool trained on ribosome profiling data and DNA sequence of translation initiation site (Shine Dalgarno sequence and ribosome binding site) to annotate protein-coding genes in the bacterial genomes. DeepRibo predicted 632 ORFs in *M. tuberculosis* excluding the annotated proteins with a prediction score >-0.5. In the investigation conducted by Smith et al. (2022), ribosome profiling techniques were employed to explore the landscape of actively translated ORFs in *M. tuberculosis*. There were 1,929 predicted small ORFs, some of which exhibited overlapping regions with annotated genes. From this, only those small ORFs that either partially or completely overlapped antisense genes or remained non-overlapping with annotated genes were considered. This yielded a subset of 1,158 small ORFs which were predominantly characterized by their short length, with a significant proportion encoding proteins of around 50 amino acids. Among these, only 757 small ORFs were of lengths > 10 amino acids.

### Conditional expression of identified ORFs

To quantify the conditional expression of the identified smORFs, strand-specific RNA-Seq data from a range of growth conditions were used. This included transcriptome sequencing of *M. tuberculosis* subjected to conditions such as redox stress (SRR14196865), low phosphate-induced stress (SRR21026187), nutrient starvation, hypoxia, and exponential growth (SRR18455925, SRR18455928 and SRR18455932). In addition, RNA-Seq data to explore the evolutionary aspects of the biofilm transcriptome (SRR23216840 and SRR23216853), assessing the RNA-Seq expression profiles of the wild-type *M. tuberculosis* H37Rv culture grown for a 48-hour duration (SRR23306196) were considered. These data were retrieved from the NCBI Sequence Read Archive (SRA) and the European Nucleotide Archive (ENA) (Leinonen et al., 2011). Downloaded fastq reads were trimmed and aligned with the reference genome (NCBI Reference Sequence: NC_000962.3) using Trimmomatic (v0.39) and Bowtie2 (v2.3.4.1), respectively (Langmead and Salzberg, 2012; Bolger et al., 2014). The read counts of these regions were obtained using MultiBamCov of bedtools and normalized as RPKM. The threshold for considering expression as significant was set above the median expression level observed for the known protein-coding regions during the exponential growth phase. smORFs were classified as expressed if their RPKM values exceeded the median RPKM value of the known protein-coding regions.

### LC-MS/MS analysis

*M. tuberculosis* LC-MS/MS data of the exponential phase and the early stationary phase (PXD000111) (Albrethsen et al., 2013) were retrieved from the PRIDE archive (https://www.ebi.ac.uk/pride/archive/) (Vizcaíno et al., 2013). To validate protein expression, *in silico* translated sequences of smORFs were given as the database in MaxQuant (v1.6.8.0) which processes LC-MS/MS raw files and searches against the protein database (Tyanova et al., 2016). smORFs of at least one unique peptide fragment with *q*-value < 0.01 were considered as expressed.

### ChIP-Seq analysis

Differential binding of RNAP and NusA for the smORFs expressing in the exponential phase was analyzed using ChIP-Seq datasets GSM1003215 and GSM1003219, respectively (Uplekar et al., 2013). Their expression levels were compared with two distinct sets of 200 randomly chosen IGRs. Additionally, ChIP-Seq data of AraC family transcriptional regulator Rv3833 and a genomic control (GSM1003222) were used as control data. These IGRs were chosen to exclude the regions between operonic genes, tRNAs, and rRNAs while maintaining similar length distributions. The fastq-dump function of the sra-toolkit-3.0.2 was used to retrieve the fastq format of the data. These were aligned to the reference genome (NC_000962.3) using bowtie2 (v 2.3.5.1). Aligned SAM files were converted to BAM files which were further sorted and indexed by samtools. From the sorted BAM files, readcounts were obtained using multiBamCov of bedtools, which were further normalized to RPKM values to represent the binding profile. Welch two sample t-test was performed to compare the binding between smORFs and random IGRs.

### G/C utilization in codons

For the smORFs, the presence of G or C bases at the first, second, and third positions of all codons was assessed. The G/C-skew was quantified by calculating the ratio of the cumulative G+C bases at the third codon position to those at the second codon position, after removing the start/stop codons. Statistical comparisons were performed using Fisher's exact test to compare the G+C base counts between the second and third positions. The statistical tests were conducted in either a one-tailed or two-tailed manner. In one-tailed tests, the null hypothesis was that the G+C base count at the third codon position would not exceed that at the second codon position.

### Gene and peptide conservation analysis

To identify the homologs of the predicted smORFs in other bacterial species, Blastn (v 2.9.0 +) (Camacho et al., 2009) with E-value <10^−4^ were used for aligning against the genomes of representative Gram-negative bacteria (*Escherichia coli* str. K-12, *Haemophilus influenzae* 86-028NP*, Klebsiella pneumoniae* MGH 78578*, Pseudomonas aeruginosa* PAO1*, Vibrio cholerae* 2010EL-1786*, Yersinia pestis* A1122*)*, Gram-positive bacteria (*Bacillus subtilis* str. 168*, Listeria monocytogenes* EGD-e*, Staphylococcus aureus* NCTC 8325*, Staphylococcus epidermidis* ATCC 12228*, Streptococcus mutans* UA159*, Streptococcus pneumoniae* 670-6B) and mycobacteria (*Mycobacterium africanum* GM041182*, Mycobacterium canetti* CIPT 140010059*, Mycobacterium tuberculosis, Mycobacterium leprae* TN*, Mycobacterium bovis* AF2122/97*, Mycobacterium avium* 104*, Mycobacteroides abscessus* ATCC 19977).

Additionally, BactPepDB database was used to test the conservation of peptides from the pool of predicted smORFs using Blastp with an E-value cutoff of <10^−2^ (Rey et al., 2014). BactPepDB is a bacterial peptide database that contains bacterial peptides of size between 10 and 80 amino acids.

### Annotation of the smORFs

For the functional annotation of the smORFs, homologous annotated proteins in other bacterial species were identified using Blastp (v 2.9.0+) against the NCBI non-redundant protein database (nr) with an E-value <10^−4^. Additionally, protein domains were identified using the InterProScan tool (https://www.ebi.ac.uk/interpro/search/sequence/) (Blum et al., 2021). Operonic gene organization in the *M. tuberculosis* H37Rv genome was obtained from MicrobesOnline Operon Predictions (http://www.microbesonline.org/operons/) (Price et al., 2005). To ensure the selection of reliable operon pairs, only those with a probability score exceeding 0.7 were included.

All the analyses were performed using in-house Python and R scripts. To generate and visualize figures, SWISS-MODEL (Waterhouse et al., 2018), Clustal Omega (Sievers et al., 2011) and PyMOL v2.5.4 (Schrödinger, LLC, 2015) were used.

## Results

### *M. tuberculosis* genome encodes numerous small peptides and proteins

Going by the arrangement of the in-frame start and stop codons along the *M. tuberculosis* genome, we identified 56,582 potential ORFs with a length cutoff of > 30 nucleotides (nts). We systematically investigated how many of these with a coding potential are indeed transcribed and translated. For this, we used a Random Forest learning algorithm that iterates over the training data and generates random decision trees and classifies the data based on the majority of decisions provided by the trees (Breiman, 2001). The training data for the prediction consisted of protein-coding sequence features, which highlight the coding capacity of a given sequence, gene expression quantified using RNA-seq, and ribosome profiling (Methods). To identify smORFs in a context-dependent manner, two sets of RNA-seq and Ribo-seq data corresponding to exponential and starvation growth conditions were used. The models were built with an accuracy of 99% for both conditions and a 5-fold cross-validation score of 0.98 and 0.99 for the exponential and starvation phases, respectively. Our final prediction resulted in 508 smORFs under exponential and 525 smORFs under starvation growth conditions, with an overlap of 338 smORFs (Supplementary Table 1). The unique proteins identified in each of these conditions suggest their potential roles in the core biological processes that transcend the impact of environmental stimuli.

The identified smORFs displayed diverse lengths, with a median size of 53 amino acids (Supplementary Figure 1A). While the majority of the smORFs identified are below 100 amino acids in size, the novel proteome pool included 143 proteins which are longer. smORFs with shorter lengths might be associated with regulatory roles while longer smORFs could potentially encode functional proteins with distinct biological roles. The categorization of the smORFs based on their genomic location showed that most of the identified regions are antisense to the known protein-coding regions (43.2%) and IGRs between two protein-coding genes (35.4%) (Supplementary Figure 1B). This implies that the bacterial gene density could be much higher than what is currently envisioned.

**Figure 1:**
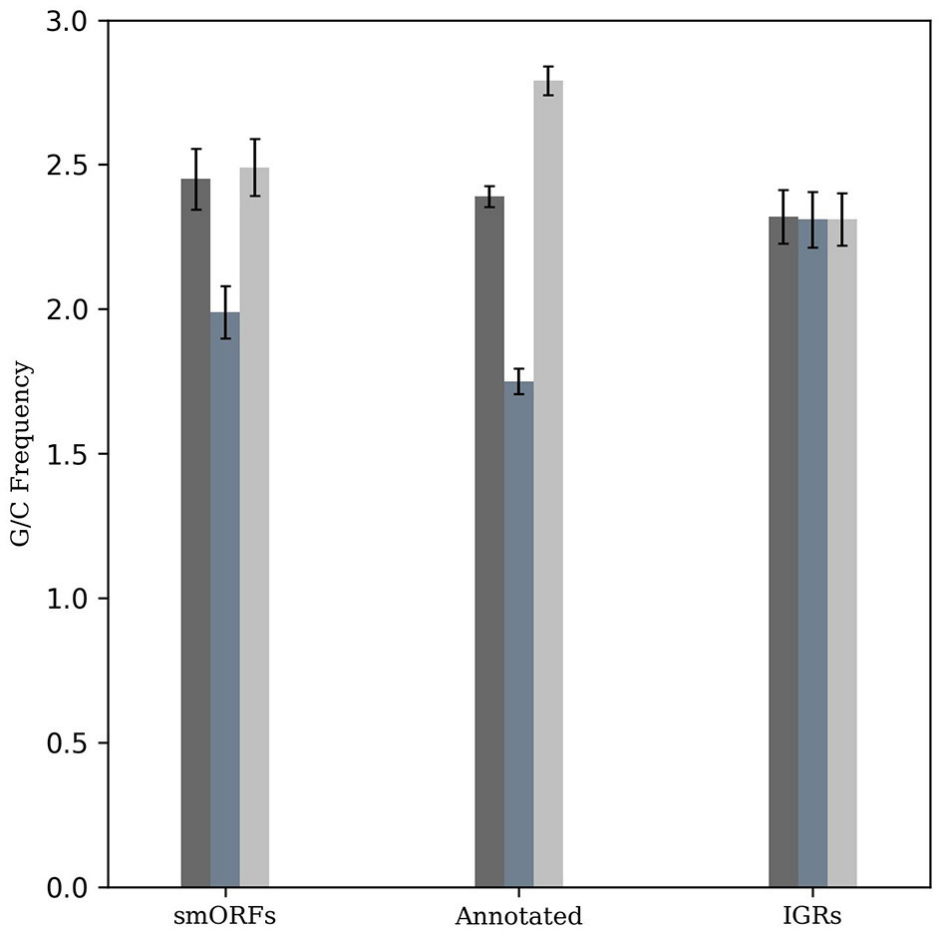
G/C-skew analysis of smORFs, annotated ORFs, and IGRs. The frequency of G+C nucleotides across different codon positions was evaluated and compared. Similar to annotated protein-coding genes, smORFs have a higher G+C content at the third codon position compared to the second position (*P* < 2.2e-16). An equal number of randomly selected IGRs are used as control.

The *M. tuberculosis* genome has a G+C content of 65.6%, exhibiting G/C-skew with higher G+C content at the third codon position compared to the second position. The G/C-skew is largely influenced by the amino acid composition of proteins, although certain amino acids may be subject to additional structural and functional constraints (Bibb et al., 1984). To evaluate whether smORF genes exhibit a similar G/C-skew pattern as observed in other *M. tuberculosis* genes, we analyzed them along with an equal number of randomly taken annotated genes and IGR sequences with a length greater than 10 amino acids. This revealed a statistically significant positive G/C-skew (Fisher's exact test, P < 2.2e^−16^; *n* = 49,470 codons) for the smORFs, in contrast to the IGRs where such skew was not observed (*P* > 0.05, *n* = 39,278 codons). The positive G/C-skew in the smORFs similar to the known protein-coding genes in *M. tuberculosis* suggests a conserved trend in codon composition in smORFs (Figure 1).

Previous attempts to identify small proteins in *M. tuberculosis* such as RanSEPs, DeepRibo, and a study by Smith et al. used either sequence features or functional data. We examined the overlap of the predicted smORFs with the small proteins identified by these methods. We observed that while 223 smORFs showed a partial or complete overlap with the small proteins identified by RanSEPs, small proteins identified by DeepRibo and Smith et al.'s work showed an overlap for 261 and 149 smORFs, respectively (Supplementary Figure 2). Interestingly, the overlap was less across these methods as well, suggesting that each of these methods captured largely distinct subsets of small proteins in *M. tuberculosis*.

Further, we tested if our prediction method including both sequence properties and functional data as features is robust in obtaining high-confidence smORFs. For this, we systematically compared the small proteins predicted by other methods with our prediction of smORFs against strand-specific RNA-Seq data available across eight distinct growth conditions (Methods). We observed that in comparison with the expression of the ORFs predicted using other mentioned tools in *M. tuberculosis*, smORFs show significantly higher expression in all the studied growth conditions (Supplementary Figure 3A). Also, the average proportion of ORFs significantly expressed in all these eight conditions was 10.68%, 40.21%, 50.36%, and 59.20% for the RanSEP, DeepRibo, Smith et al.'s approach, and smORFs, respectively (Supplementary Figure 3B). This demonstrates consistently higher levels of expression of the smORFs identified by our approach across all the growth conditions in comparison to other prediction methods, highlighting the benefit of integrating sequence and functional data while predicting smORFs in the bacterial genomes.

### Functional analysis of the novel proteome of *M. tuberculosis*

The functional data comprising of RNA-Seq and Ribo-Seq used for the prediction included exponential and starvation growth conditions. Further, we studied the expression of smORFs in eight diverse growth conditions to test their context-dependent expression and the implications thereof (growth conditions listed in Methods) (Supplementary Table 2, Supplementary Figure 4). The condition-specific expressions could be indicative of *M. tuberculosis* adapting to different environmental stresses encountered during infection. Of the 695 small proteins, 133 were expressed across all the tested conditions, indicating their potential functional relevance across diverse conditions (Supplementary Figure 5). For instance, the transmembrane domain-containing protein MTB_ORF_12681 shows expression in all the growth conditions. This protein is conserved in *M. bovis* Mexico and *M. leprae* str. as given in BactPepDB. Additionally, 55 smORFs exhibited expression in at least one growth condition, suggesting condition-specific roles for these proteins. For instance, MTB_ORF_15161 predicted under the starvation phase showed significant expression levels in RNA-seq nutrient-starved growth conditions. MTB_ORF_15161 is conserved in different mycobacterial species and was also detected in LC-MS/MS analysis. Another example is MTB_ORF_18442, which was predicted exclusively for the starvation phase and detected in LC-MS/MS analysis. MTB_ORF_18442 shows significant expression in both nutrient starvation and hypoxic growth conditions. Also, MTB_ORF_19293 identified in the starvation phase has significant expression in redox stress and low phosphate growth conditions. MTB_ORF_19293 is a PIN domain-containing protein homologous to the VapC family toxin (Type II toxin-antitoxin system) protein in *Gordonia mangrovi, Cryobacterium serini*, and different mycobacterial species. *M. tuberculosis* genome has numerous toxin-antitoxin systems, notably the VapBC family with 50 members (Chauhan et al., 2022), which involve PIN-domain proteins known for their ribonuclease activity (Arcus et al., 2005). These findings highlight the dynamic and context-dependent nature of small protein expression in *M. tuberculosis*, suggesting their involvement in specific physiological processes.

**Figure 2:**
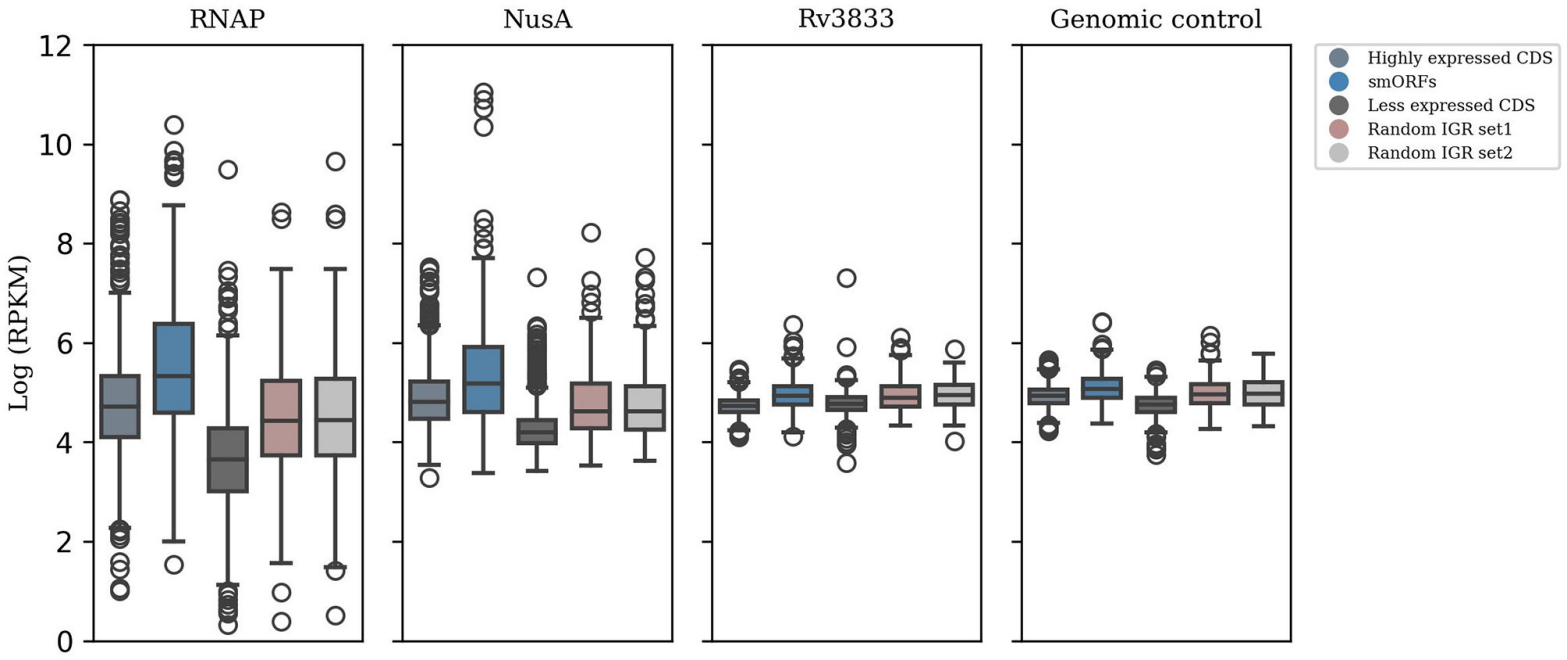
ChIP-seq data analysis to test smORF transcription. Significant binding of RNAP and NusA in the genomic locations encoding smORFs suggests active transcription in these regions, which is comparable to binding in the highly expressed genic regions in *M. tuberculosis* (P-value < 0.05). This is contrasted with their binding to randomly selected IGRs and less expressed genic regions. Additionally, genomic control and ChIP-seq data of the transcription factor Rv3833 were used as control samples.

Further, we attempted to validate the expression of smORFs by testing the binding of transcription machinery in these genomic regions. RNAP stands as the primary enzyme responsible for transcribing RNA from a DNA template. Another member of the transcription complex is NusA, which dynamically associates with RNA polymerase and impacts elongation rates (Vogel and Jensen, 1997; Uplekar et al., 2013). We analyzed ChIP-Seq data of RNAP and NusA for the 508 smORFs identified in the exponential growth phase. In addition, genomic control and transcription factor Rv3833 data were included as control samples. We observed that the smORFs were significantly bound by RNAP and NusA compared to random IGRs (P-value < 0.05) (Figure 2). Therefore, the transcription machinery appears to be strongly coupled to the genomic regions encoding smORFs, indicating transcriptional activity in these regions.

To assess the functional relevance of the smORFs, we integrated biological data arising from LC-MS/MS, protein domains and families, homologs identified in the NCBI non-redundant protein database (nr), and sequence similarity results obtained from Blastn against bacterial genomes. Using Interpro and its member databases (described in the Methods section), we predicted conserved protein domains for 257 smORFs. Also, Blastp against the nr database showed 333 ORFs as the homologs of smORFs that are conserved in different bacterial genera and species (Supplementary Figure 6). Conservation of the smORFs in some of the Gram-negative, Gram-positive, and mycobacterial genomes (listed in Methods) evaluated using Blastn identified 693 homologs. While only 1 homolog for the smORFs was found in both the studied Gram-positive bacteria and Gram-negative bacteria, 681 showed conservation in the pathogenic *M. tuberculosis* and other members of the *M. tuberculosis* complex (MTBC) such as *M. africanum, M. canetti*, and *M. bovis*. Importantly, LC-MS/MS analysis showed the translated products for the 25 smORFs, many of which also have conserved protein domains. Since the majority of the smORFs were of length shorter than 50 amino acids, we also scanned smORFs through the BactPepDb with default search parameters and obtained the hits for 225 smORFs. For instance, MTB_ORF_36476 is conserved in *Nocardia cyriacigeorgica* GUH-2, *Rhodococcus jostii* RHA1, *Saccharomonospora viridis* DSM 43017, and other bacterial species. Results from all these analyses are provided as a comprehensive table (Supplementary Table 3). Below, we discuss a few examples that show agreement from more than one of the above-mentioned analyses.

**Figure 3:**
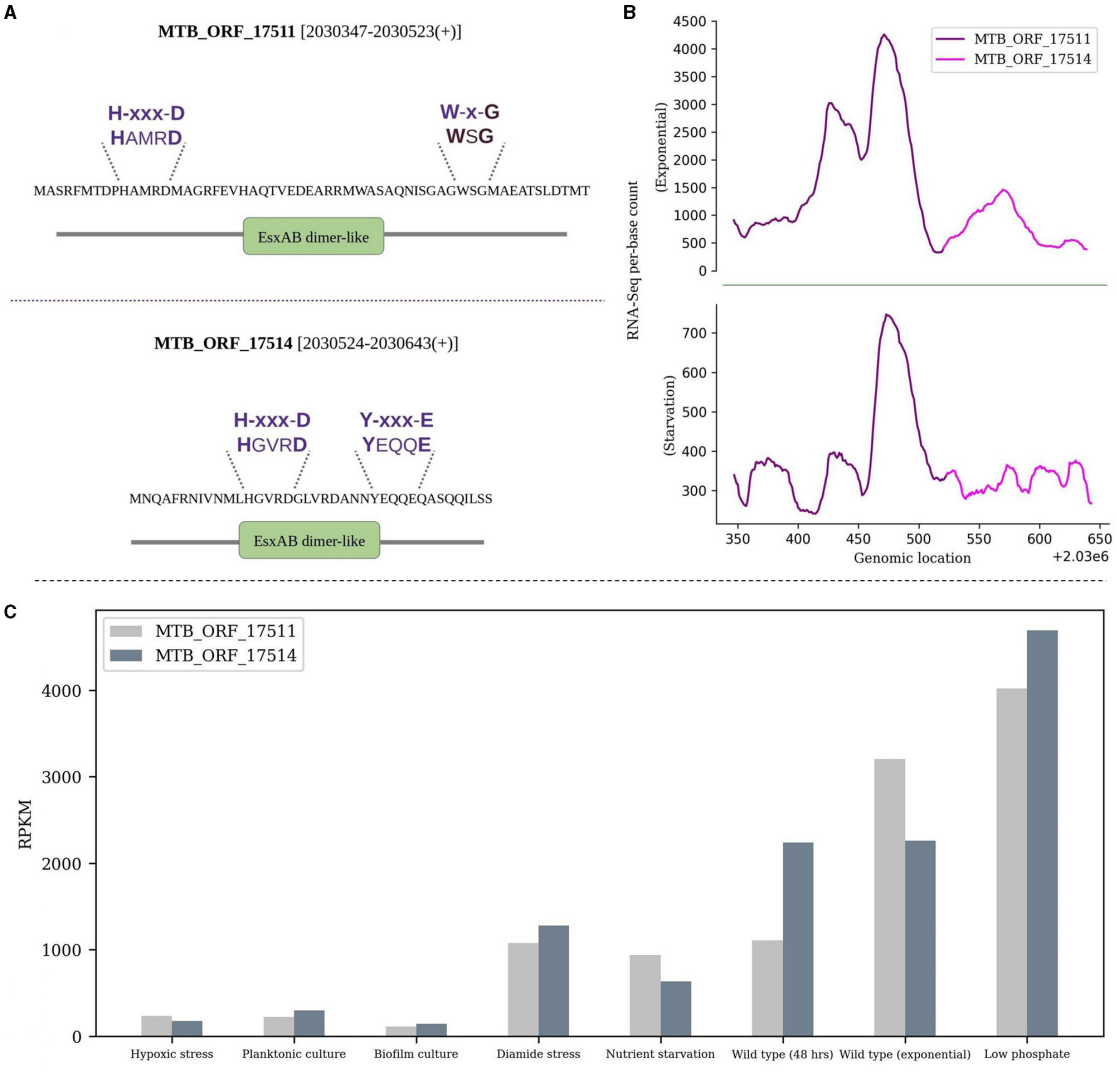
Sequence and expression analysis of MTB_ORF_17511 and MTB_ORF_17514. **(A)** Structural features and conserved motifs of the smORFs. MTB_ORF_17511 is characterized by the H-xxx-D and W-x-G motifs, while MTB_ORF_17514 exhibits the H-xxx-D and Y-xxx-E motifs. **(B)** Displays RNA-Seq expression levels of MTB_ORF_17511 and MTB_ORF_17514 during the exponential and starvation growth phase, and **(C)** Expression of MTB_ORF_17511 and MTB_ORF_17514 quantified from RNA-Seq data from eight different growth conditions. Relatively high expression levels are observed in low phosphate conditions.

i) MTB_ORF_47982 conserved in *M. bovis* and *M. canettii* was found to have a Class A radical SAM methyltransferase domain. The Class A methyltransferase domain catalyzes methylation of adenosine in RNA and contains two conserved Cys residues for catalysis, utilizing SAM as both a methyl donor and a source of the activating radical (Fujimori, 2013). Proteins with radical SAM core domains use a methylene radical from methylated cysteine residues (Zhang et al., 2012). They have a CxxxCxxC motif crucial for coordinating [4Fe-4S]2+/1+ clusters (Sofia et al., 2001) and a structure resembling a TIM barrel, similar to TIM barrel enzymes (Davis et al., 2017). This smORF has a conserved cysteine motif and also showed an overlapping Aldolase class I domain, characterized by a TIM β/α barrel structure (Supplementary Figure 7A). Another smORF, MTB_ORF_68067 contains a homeodomain-like domain (PF13384) that binds DNA through a helix-turn-helix (HTH) structure (IPR009057) and belongs to Winged helix-like DNA-binding domain superfamily (Supplementary Figure 7B). MTB_ORF_68067 is homologous to transposase (helix-turn-helix domain-containing protein) of different mycobacterial species and actinobacterial species such as *Actinomycetota bacterium, Kribbella soli, Blastococcus endophyticus*, and *Mycobacterium avium*.
ii) The WXG100 protein superfamily, exemplified by *M. tuberculosis* ESAT-6 protein, is associated with the Type VII secretion system (T7SS). The key characteristics include the “Trp-Xaa-Gly (WXG)” motif along with crucial motifs YxxxD/E and HxxxD/ExxhxxxH (Poulsen et al., 2014). MTB_ORF_1751 and MTB_ORF_17514 are adjacently located in the IGR between *Rv1791* and *Rv1793* on the positive strand. Both the smORFs feature an EsxAB-dimer-like domain and belong to the ESAT-6-like superfamily (IPRO36689). ESAT-6 is an important virulence factor and a major secreted protein of the ESX-1 secretion system in *M. tuberculosis* (Sreejit et al., 2014; Osman et al., 2022). MTB_ORF_17511 contains a conserved W-x-G motif, while MTB_ORF_17514 features a conserved YxxxD/E motif. Additionally, both smORFs exhibit a conserved HxxxD motif. It's worth noting that these ORFs, although overlap with a pseudogene *Rv1792*, show significant RNA-Seq expression. Also, their presence was exclusively detected during the exponential growth phase in LC-MS/MS analysis. The expression levels in RNA-Seq and the respective domains and motifs of these smORFs are depicted in Figures 3A, B. Interestingly, the gene that is present next to MTB_ORF_17514 is *Rv1793*, an ESAT-6-like protein EsxN of the ESX-1 secretion system. There is a possibility that these three ORFs collectively form an operon. It is reported that low phosphate conditions can induce both the ESX-5 and ESX-1 secretion systems (Elliott and Tischler, 2016). RNA-Seq analysis revealed favorable expression patterns for both smORFs, with detectable expression across all eight conditions and notably elevated expression levels in the specific context of low phosphate conditions (Figure 3C). Also, MTB_ORF_17514 and MTB_ORF_17511 exhibit conservation across various mycobacterial species, including *M. kansasii, M. canettii, M. simulans*, and also in *E. coli*.
iii) The Nudix superfamily, widely distributed in eukaryotes, bacteria, archaea, and viruses, primarily consists of pyrophosphohydrolases that target substrates with the general structure NDP-X and have significance in diverse biological systems (McLennan, 2006). MTB_ORF_61001, located antisense to *Rv1499*, contains a partially conserved NUDIX box motif and NUDIX domain in it which is characterized by the conserved loop-helix-loop structure (Mildvan et al., 2005) (Supplementary Figure 8). This smORF was also identified in LC-MS/MS analysis.
iv) In *M. tuberculosis*, cutinase-like proteins (CULPs) show unique enzymatic activities, such as esterase and lipase functions, despite their cutinase resemblance. CULPs are characterized by α/β hydrolases featuring a conserved pentapeptide motif (G-[YF]-S-[QL]-G) (West et al., 2009). MTB_ORF_35316 and MTB_ORF_35318 have α/β hydrolase and cutinase domains. Both smORFs are conserved along many mycobacterial species such as *M. kansasii* and *M. canettii*. Moreover, MTB_ORF_35318 also has a conserved G-[YF]-S-[QL]-G motif (Supplementary Figures 9A, B).
v) MTB_ORF_57155 contains a BFD-like [2Fe-2S] binding domain, a feature also found in bacterioferritin-associated ferredoxin (Bfd). Bfd plays a key role in bacterial iron homeostasis by aiding in the release of Fe3+ stored in BfrB (Bacterioferritin). As a [2Fe-2S]-protein, Bfd interacts with BfrB to facilitate the mobilization of iron (Wijerathne et al., 2018). Bfd is a NIFU-like [2Fe-2S] protein, characterized by a shared conserved cysteine pattern (C-X-C-X32-C-X2-C), suggesting a common modular domain for binding [2Fe-2S] clusters (Garg et al., 1996). The sequence alignment of MTB_ORF_57155 to (2Fe-2S)-binding proteins of *M. lepra* and *E. coli* is shown in Supplementary Figure 10, highlighting the conserved cysteine residues. MTB_ORF_57155 is homologous to the (2Fe-2S)-binding protein of *Rhoodococcus phenolicus* and is conserved in many other mycobacterial species such as *M. palustre, M. porcinum, M. kubicae, M. aquaticum*, and *M. canettii*.

Overall, these findings provide insights into the evolutionary conservation, expression patterns, and potential functional roles of the smORFs in *M. tuberculosis*.

### Genomic and functional organization of the smORFs

#### Dual function sRNAs

Certain bacterial sRNAs encode functional peptides in addition to modulating gene expression through base-pairing with mRNAs (Thomason and Storz, 2010). This introduces complexity to bacterial gene regulation, as these dual-function sRNAs simultaneously govern post-transcriptional control and diversify the proteome of an organism (Gimpel and Brantl, 2017). We observed such a potential dual-function for 9 smORFs which originate from the reported sRNA-encoding regions in *M. tuberculosis* (Supplementary Table 4A). For instance, MTB_ORF_64551 (90 amino acids) spanning the sRNA MTS0903 (Arnvig et al., 2011) is conserved as a protein in *M. riyadhense* and *Streptomyces* sp. XY431 with 86% and 41% identity, respectively. Likewise, MTB_ORF_12647 (35 amino acids) which is homologous to the MurA protein of *M. kansaii* overlaps with the sRNA RVnc0021 (*mcr3*) which is reported to have an important role in early and late hypoxia in the presence of dextrose (Del Portillo et al., 2019). Notably, MTB_ORF_12647 showed significant expression in all the 8 studied growth conditions. Moreover, the prediction tools DeepRibo and RanSEPs also partially/completely predicted MTB_ORF_64551 and MTB_ORF_12647 as potential small proteins, further supporting their dual functionality. Another smORF MTB_ORF_46462 (19 amino acids), which is conserved as a peptide in the BactPepDB in different mycobacterial species and also predicted as an ORF by DeepRibo and RanSEPs, overlaps with a 5′UTR sRNA, referred as “candidate 1502” (Miotto et al., 2012). This sRNA is located upstream of the gene *ilvB* which is part of the isoleucine-valine operon, suggesting that sRNA might be involved in an attenuator system that regulates the synthesis of valine and isoleucine in mycobacteria. MTB_ORF_46462 is significantly expressed in 7 out of 8 studied growth conditions. These findings, therefore, suggest a potential dual functionality of a few smORFs, where they act as sRNAs by engaging in antisense/complementary interactions, while also participating in metabolic pathways as short peptides.

#### Co-regulated gene clusters

**Figure 4:**
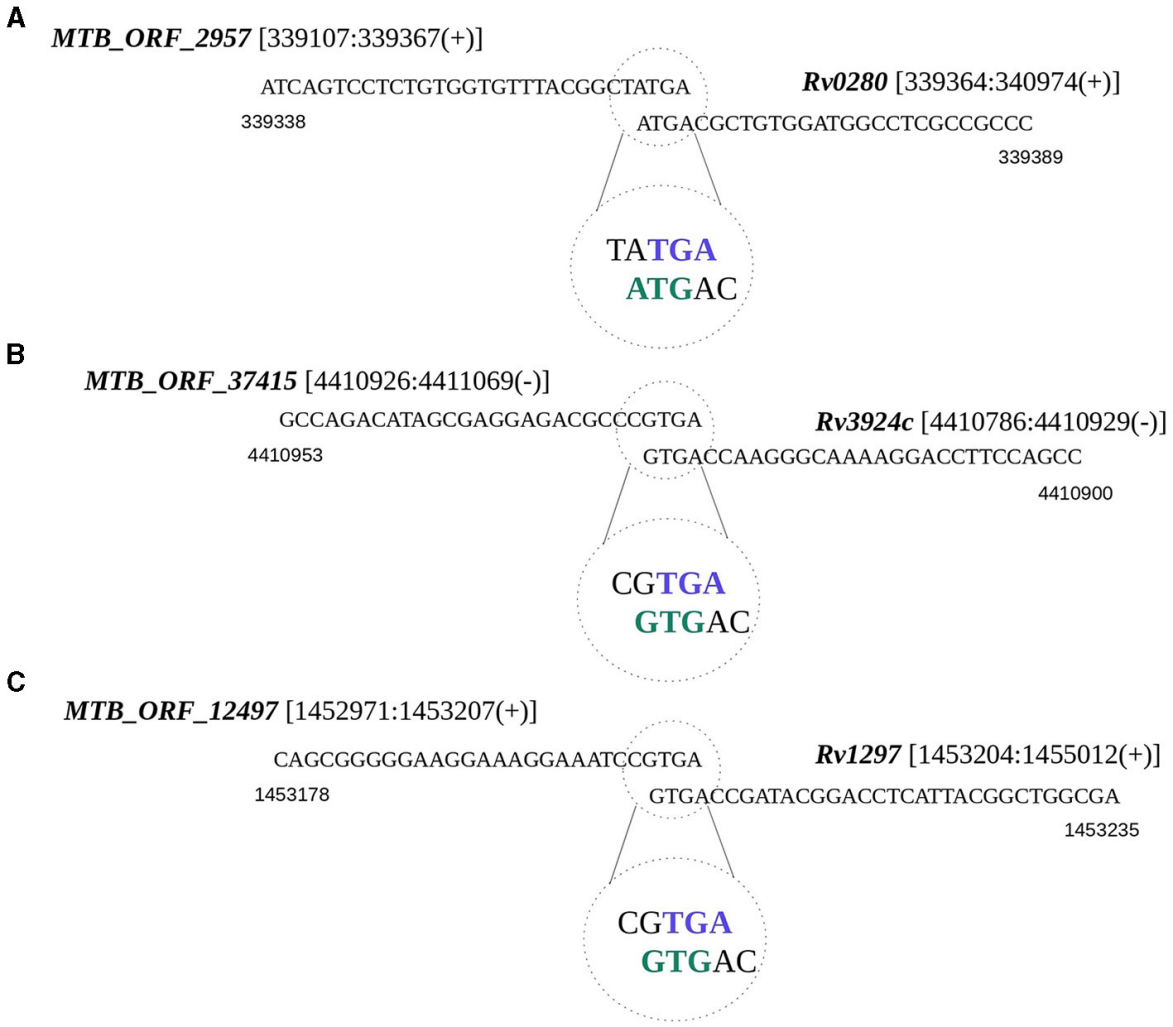
Translational coupling revealed by smORF gene sequences. The overlapping start or stop codons between smORFs and the annotated gene is regarded as a potential translation coupling. **(A)** Coupling between *MTB_ORF_2957* and *Rv0280*; **(B)** Coupling between *MTB_ORF_37415* and *Rv3924c*; and **(C)** Coupling between *MTB_ORF_12497* and *Rv1297*.

Translational coupling is a phenomenon observed in prokaryotes, particularly archaea and bacteria, where the translation of a downstream gene is dependent on the translation of the upstream genes (Huber et al., 2019). To test whether any of the smORFs exist as coupled ORFs, we examined the occurrences of overlapping start or stop codons between smORFs and any annotated gene in *M. tuberculosis*. We identified 50 such instances, of which 16 appear to be within the operons (Supplementary Table 4B). The start or stop codons of these smORFs are situated in a manner that they potentially overlap with the start or stop codon of the neighboring annotated gene. For example, MTB_ORF_2957 with a membrane binding domain is coupled with a PPE protein-encoding *Rv0280* (Figure 4A). Many PPE proteins in *M. tuberculosis* are known to be associated with the cell membrane and have membrane-binding properties (Mitra et al., 2017). MTB_ORF_2957 is homologous to the PPE family protein of *M. tuberculosis* var. africanum. In another instance, MTB_ORF_37415 is coupled with the start codon of *rpmH* (*Rv3924c*), and the presence of this peptide was also identified in the LC/MS-MS study (Figure 4B). Another example is MTB_ORF_12497, which is coupled with the start codon of *rho* (*Rv1297*) (Figure 4C). MTB_ORF_12497 shows conservation across *M. canettii, M. bovis*, and *M. Avium*, and this coupling between the two genes appears to be conserved. Further exploration of these diverse genomic organizations of smORFs in the context of annotated proteins could provide valuable insights into the complex regulatory and functional aspects of smORFs in *M. tuberculosis*.

Upstream open reading frames (uORFs) are the small protein-coding regions in mRNA that precede the main coding region of a gene. Bacterial uORFs, known as leader peptides, can stall ribosomes during translation, influencing mRNA production and downstream gene expression. These uORFs can also be coupled to the translation of main protein-coding ORFs, influencing the overall translation efficiency of the mRNA (Dever et al., 2020; Morris and Geballe, 2000). We scanned the 5′UTRs of the annotated protein-coding genes in *M. tuberculosis* to test for the presence of any of the identified smORFs. For this, we used the available genome-wide transcription start sites (TSS) in *M. tuberculosis* (Cortes et al., 2013) and identified 58 and 77 putative uORFs during exponential and starvation growth conditions, respectively (Supplementary Table 4C). For example, MTB_ORF_67572, predicted in both exponential and starvation growth conditions, is located in the 5′UTR of *Rv0813c*. Rv0813c belongs to a family of bacterial FABP-like proteins and may have a role in the recognition, transport, and/or storage of small molecules in the bacterial cytosol (Shepard et al., 2007). MTB_ORF_67572 has a transmembrane domain, implying its potential function as a membrane-bound protein. Notably, this smORF is conserved as a membrane protein in Gram-positive bacterial species, including *Gordonia jacobaea, Rhodococcus aetherivorans*, as well as various mycobacterial species such as *M. kansasii* ATCC 12478, and *Mycolicibacterium wolinskyi*. These findings suggest a promising avenue for further research into the regulatory roles of these putative smORFs as uORFs in *M. tuberculosis*.

**Figure 5:**
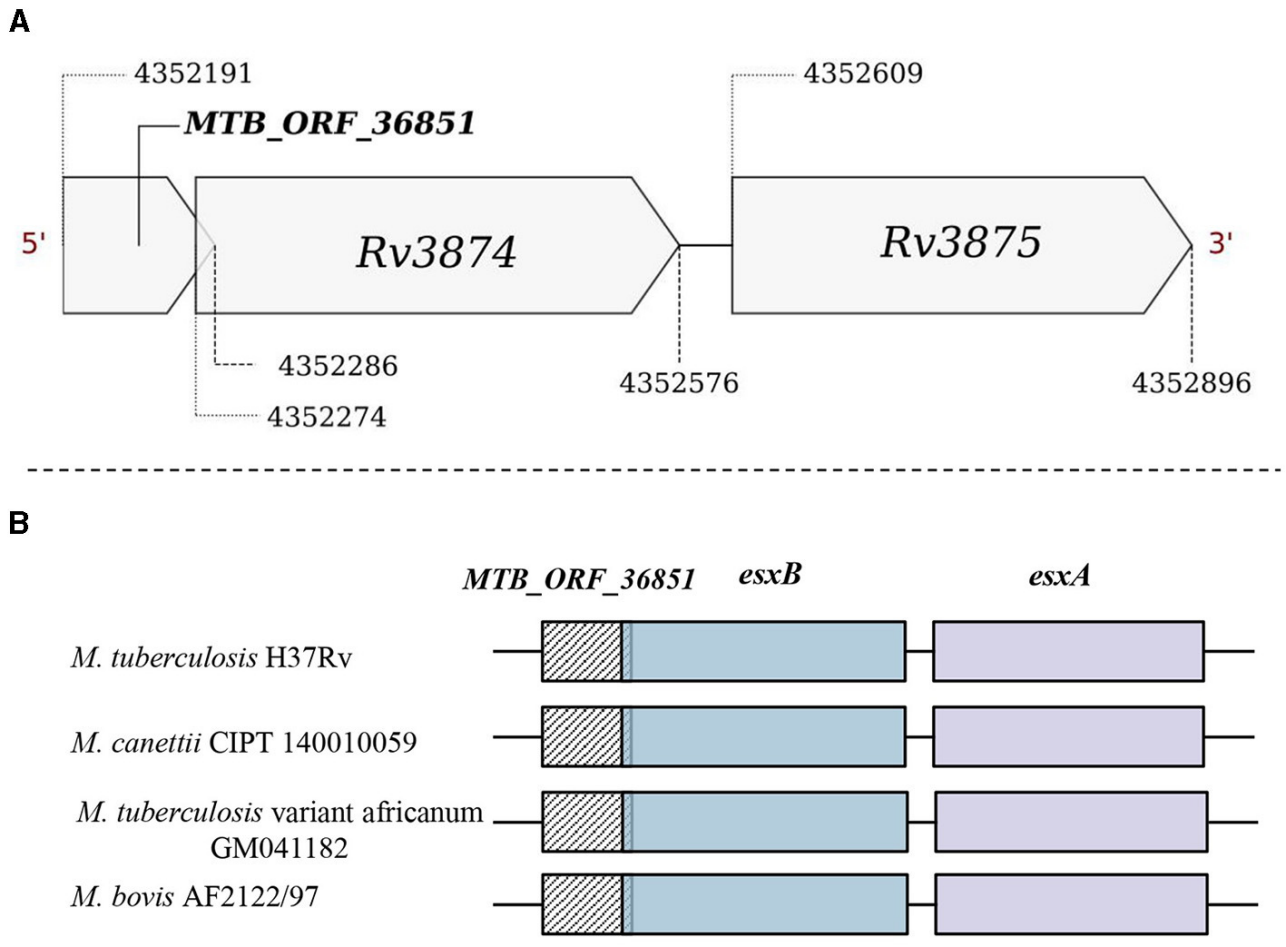
Genomic location of MTB_ORF_36851. **(A)** MTB_ORF_36851 appears to be in an operon along with the genes *Rv3874* and *Rv3875* and **(B)** Conservation of gene synteny involving MTB_ORF_36851 within the suggested operon structure across mycobacterial species, including *M. canettii* CIPT 140010059 (4422565-4423270), *M. tuberculosis* var. africanum GM041182 (4329924-4330629), and *M. bovis* AF2122/97 (4292923-4293628).

Operon gene organization enables the functional grouping of the genes to achieve rapid adjustments in response to changing environments, enhancing bacterial adaptability and survival (Ralston, 2008). We examined if any of the smORFs could be part of the defined operonic structures, and identified 65 smORFs located within the operonic regions in *M. tuberculosis* (Supplementary Table 4D). For example, MTB_ORF_54512 is part of an operon with *Rv2159c, Rv2160A*, and *Rv2160c* (Supplementary Figure 11). MTB_ORF_54512 has a Tetracyclin repressor-like, C-terminal domain and overlaps with an annotated pseudogene *Rv2160c*. TetR controls the production of TetA, a protein located in the cell membrane responsible for expelling tetracycline antibiotics from the cell, preventing them from binding to ribosomes and disrupting protein synthesis (Kisker et al., 1995). In another example, MTB_ORF_16006 is an integral part of an operon comprising *Rv1641, Rv1642, Rv1643*, and *Rv1644* (Supplementary Figure 12). The proteins in this operon share functions closely related to ribosome assembly and protein synthesis. Notably, MTB_ORF_16006 features a membrane-binding domain and is conserved as a peptide in *M. leprae*. In a different instance, MTB_ORF_36851 is part of the ESX protein operon *Rv3874-Rv3875* (Figure 5A). MTB_ORF_36851 has a 12bp overlap with *Rv3874* and was also detected in LC/MS-MS analysis. This smORF demonstrates conserved gene synteny along with the smORF in *M. canettii, M. bovis*, and *M. tuberculosis* variant africanum (Figure 5B). Moreover, smORFs MTB_ORF_16006 and MTB_ORF_36851, which are positioned at the start of their respective operons and are smaller in size compared to the other operonic genes, might function as uORFs. These smORFs demonstrate significant expression levels across all eight tested growth conditions, emphasizing their functional importance (Methods). These findings suggest that smORFs within operons could play a crucial role in *M. tuberculosis* adaptability and survival, particularly concerning key cellular processes and antibiotic resistance.

## Discussion

Small proteins have come to light either by recognizing that the mutations in IGR are attributed to unannotated small protein genes or through the revelation that some sRNAs encode small proteins (Storz et al., 2014). Most genome projects employed a 100 amino acid residue threshold to avoid erroneous predictions, resulting in the under-representation of small proteins (Su et al., 2013). Traditional protein discovery methods also faced challenges in small protein prediction and validation. However, these seemingly trivial molecules have gained recognition as they play important roles in processes such as cell signaling, metabolism, and growth (Su et al., 2013). Since small proteins play a significant role in bacterial pathogenesis and virulence, it is important to define their function and interactions.

We identified small proteins in *M. tuberculosis* using a comprehensive approach that incorporated sequence features, RNA-Seq, and Ribo-Seq data that are publicly available. The data underwent rigorous training and testing on selected features, resulting in the prediction of 695 smORFs. To assess whether combining sequence features along with high-throughput functional data such as RNA-Seq and Ribo-Seq generates robust predictions, we systematically compared the small proteins sourced by other available methods and tools with our prediction of smORFs. For this, we tested the proportion of small proteins that are expressed in independent RNA-Seq data and noted that smORFs show significant expression across tested growth conditions compared to those generated by previous methods. The elevated expression levels observed in smORFs underscore the robustness of our approach, which integrates both sequence characteristics and expression data for ORF prediction. Importantly, the genomic regions encoding these smORFs show statistically significant binding of RNAP and NusA in contrast to randomly selected IGRs, thereby providing strong evidence for their expression. Subsequently, a thorough homology search against the nr database was conducted to identify conserved protein domains in the smORFs. Further, a comprehensive exploration included testing their conservation across genomes and peptide database, detection in the LC-MS/MS data, and studying their genome context features such as operon organization, coupling with annotated genes, and uORF analysis. Remarkably, several smORFs met most of the aforementioned criteria, making them intriguing candidates for further exploration.

MTB_ORF_17511 and MTB_ORF_17514 collectively encompass a pseudogene. Although a pseudogene region is not expected to be transcribed, it's noteworthy that the smORFs not only possess the conserved EsxAB-dimer-like domain featuring WXG motifs but also exhibit expression in all the growth conditions studied using RNA-Seq data. Similarly, MTB_ORF_54512 overlaps with a pseudogene and displays a C-terminal domain resembling that of a Tetracyclin repressor. Another smORF, MTB_ORF_35318 situated on the positive strand of *M. tuberculosis* is 208 amino acids long. This smORF is located at 5′UTR of *Rv3724B* and has a conserved cutinase domain and motif. Moreover, the presence of a BFD-like [2Fe-2S] binding domain in MTB_ORF_57155, similar to the bacterioferritin-associated ferredoxin (BFD), is of particular importance. BFD, known for its shared conserved cysteine pattern, suggests a common modular domain for binding [2Fe-2S] clusters. The observed homology of MTB_ORF_57155 with (2Fe-2S)-binding proteins in various mycobacterial species underscores its potential functional relevance.

Many smORFs were identified to serve a dual role as sRNAs. For instance, MTB_ORF_64551, which is reported to function as an sRNA MTS0903, is conserved as a protein in different mycobacterial species. Besides these smORFs, we identified multiple smORFs that could be (i) a part of an operon, (ii) function as an upstream ORF or (iii) have their start/stop codon coupled with the annotated ORFs. For example, MTB_ORF_36851, situated upstream of an operon consisting of *Rv3874* and *Rv3875*, is conserved in different mycobacterial species along with the whole operon. Consequently, it may function as an integral component of this operon or act as an uORF. Also, a membrane-biding domain-containing MTB_ORF_2957 is coupled with a PPE protein *Rv0280*. This observation agrees with known characteristics of PPE proteins in *M. tuberculosis* which are often associated with the cell membrane. Furthermore, the homology of MTB_ORF_2957 to a PPE family protein in *M. tuberculosis* var. africanum underscores its conservation and functional relevance in the broader context of mycobacterial species.

These instances provide valuable information for further investigation into the functional roles of smORFs in *M. tuberculosis* and other related organisms. Further experimental validation is necessary to understand the mechanism of action and functional significance of the smORFs. Understanding the roles of small proteins, particularly in stress conditions, could pave the way for developing novel antimicrobial strategies. Future research directions may include functional studies of these small proteins, exploring their roles in various strains or stress conditions, and comparative analyses to understand their conservation and diversity across different mycobacterial species and other bacteria. These efforts could provide crucial insights into the pathogenicity mechanisms and adaptive strategies of *M. tuberculosis* and related bacteria, potentially leading to new therapeutic approaches.

Overall, our findings shed light on the potential importance of small proteins in mycobacterial biology and provide a foundation for future research in this area.

## Data availability statement

The original contributions presented in the study are included in the article/Supplementary material, further inquiries can be directed to the corresponding author.

## Author contributions

PS: Data curation, Formal analysis, Methodology, Validation, Visualization, Writing – original draft. RB: Data curation, Formal analysis, Methodology, Writing – original draft. SH: Conceptualization, Formal analysis, Methodology, Supervision, Writing – review & editing.

## Supplementary material

Supplementary Figure 1 **(A)** Distribution of smORF lengths, and **(B)** Genome-wide distribution of smORFs. For each category, the number of smORFs and their respective percentages are mentioned in the figure.

Supplementary Figure 2 Venn diagram illustrating shared small protein predictions among the current study and the previous reports namely, RanSEPs, DeepRibo, and Smith et al.'s Riboret approach.

Supplementary Figure 3 Assessing the performance of different small protein prediction methods by evaluating ORF expression in diverse growth conditions. **(A)** The percentage of small proteins crossing the median expression level of annotated CDS regions in each of the eight individual RNA-Seq conditions is calculated and represented in the Y-axis, **(B)** The results from **(A)** are averaged to represent the consolidated performance for each method.

Supplementary Figure 4 Circular plot illustrating context-dependent expression of the smORFs in 8 distinct RNA-Seq data, ordered from the outermost to the inner circle as specified in Supplementary Table 2. The plot is generated using the Circos tool (Krzywinski et al., 2009).

Supplementary Figure 5 Each bar represents the count of smORFs that are significantly expressed in a varying number of conditions. The last column indicates that 133 smORFs are significantly expressed in all eight conditions.

Supplementary Figure 6 Distribution of the hits obtained for the smORFs from Blastp against the nr database across bacterial genera. The Y-axis denotes the number of Blastp hits.

Supplementary Figure 7 Protein domains highlighted in the identified smORFs. **(A)** Presence of conserved Aldolase class I and Radical SAM domains, along with a conserved **C**xxx**C**xx**C** motif within MTB_ORF_47982, and **(B)** Winged helix-like DNA-binding domain superfamily in MTB_ORF_68067.

Supplementary Figure 8 Illustration of MTB_ORF_61001 with a NUDIX domain in protein sequence and structure (highlighted in teal). The model was built using SWISS-MODEL taking A0A7G7DNV8 (Alphafold) as a template.

Supplementary Figure 9 Representation of the **(A)** Cutinase and AB_hydrolase domains in MTB_ORF_35316; **(B)** Cutinase and AB_hydrolase domains in MTB_ORF_35318, including a conserved GxSxG motif.

Supplementary Figure 10 **(A)** A BFD-like [2Fe-2S] domain identified within MTB_ORF_57155, and **(B)** Sequence alignment between BFD protein of *E. coli* (E.c.) (CAD6001291.1), (2Fe-2S)-binding protein of *M. leprae* (M.l.) (WP_081439367.1), and MTB_ORF_57155 (MORF_57155), generated using CLUSTAL O (1.2.4). The conserved cysteine residues are highlighted with green bars.

Supplementary Figure 11 *MTB_ORF_54512* within an operon that includes *Rv2159c, Rv2160c*, and *Rv2160A*. A Tetracycline repressor-like C-terminal domain is found in MTB_ORF_54512.

Supplementary Figure 12 Representation of *MTB_ORF_16006* within an operon that includes the genes *Rv1641, Rv1642, Rv1643*, and *Rv1644*.

Supplementary Table 1 The genomic locations of smORFs and the growth condition in which they were predicted.

Supplementary Table 2 The accession numbers and the condition descriptions for the 8 different RNA-Seq data used.

Supplementary Table 3 Compilation of data obtained from multiple analyses. **(A)** Identified protein domains in the smORFs, **(B)** Blastp hits against nr database obtained for the smORFs, **(C)** Blastp hits using Bacterial Peptide Database (BactPepDb), **(D)** Results of the Blastn analysis performed against select Gram positive, Gram negative and mycobacterial genomes, and **(E)** Outcomes of the LC/MS-MS analysis using Maxquant.

Supplementary Table 4 smORF genomic organization along with other annotated genes and sRNAs. **(A)** Percent overlap between smORFs and experimentally validated sRNAs, **(B)** Coupling and basepair overlap between smORFs and the corresponding annotated genes, **(C)** Presence of smORFs within the Transcription Start Site (TSS) region of the annotated genes, and **(D)** smORFs as part of the operonic structures.
