# Supplementary figures and images for: Integrated sequence and -omic features reveal novel small proteome of Mycobacterium tuberculosis
Source: Front Microbiol. 2024 May 15;15:1335310. doi: 10.3389/fmicb.2024.1335310 (PMC11133741; doi:10.3389/fmicb.2024.1335310)

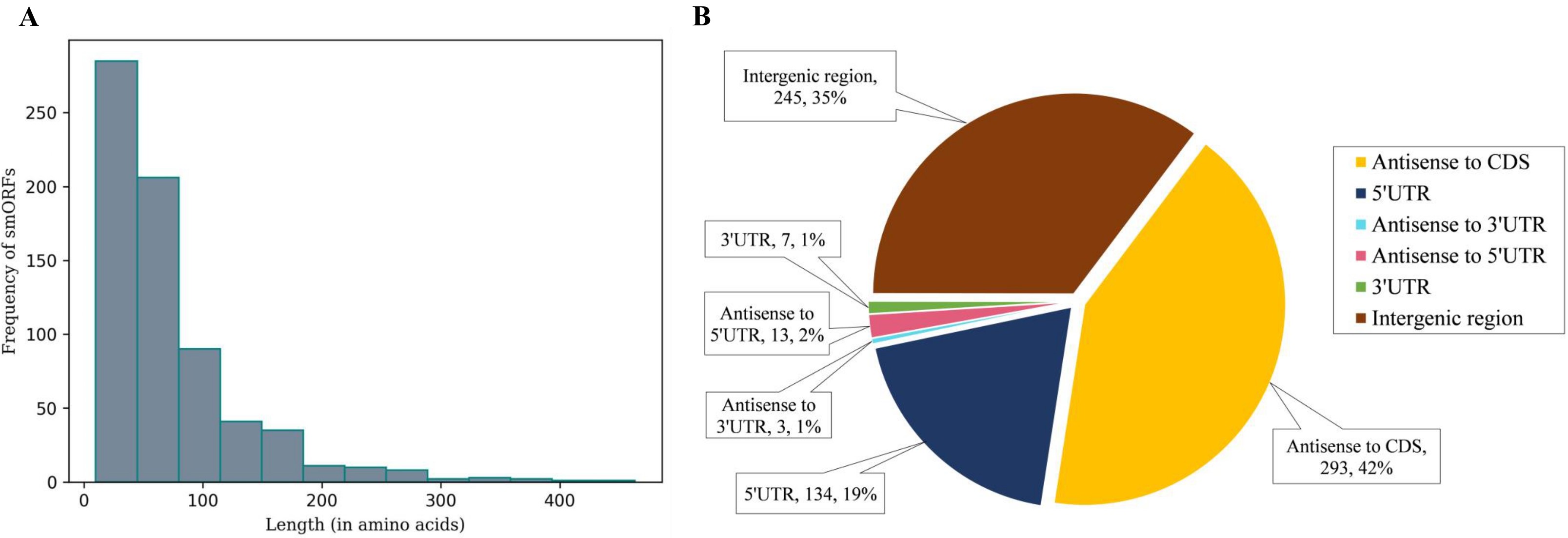

Supplement: Supplementary Figure 1 — (A) Distribution of smORF lengths, and (B) Genome-wide distribution of smORFs. For each category, the number of smORFs and their respective percentages are mentioned in the figure. [file Image_1.JPEG]

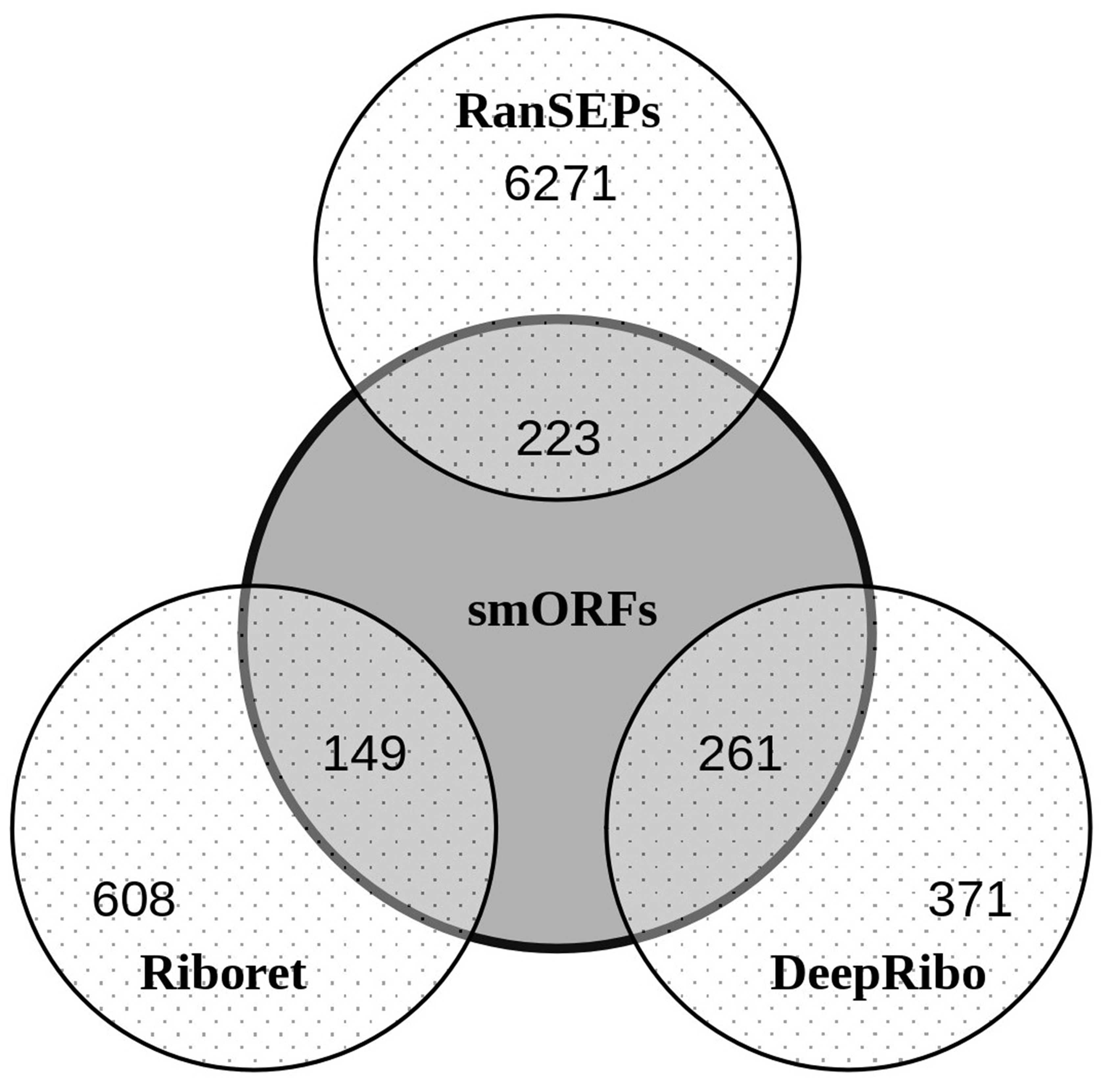

Supplement: Supplementary Figure 2 — Venn diagram illustrating shared small protein predictions among the current study and the previous reports namely, RanSEPs, DeepRibo, and Smith et al.'s Riboret approach. [file Image_2.JPEG]

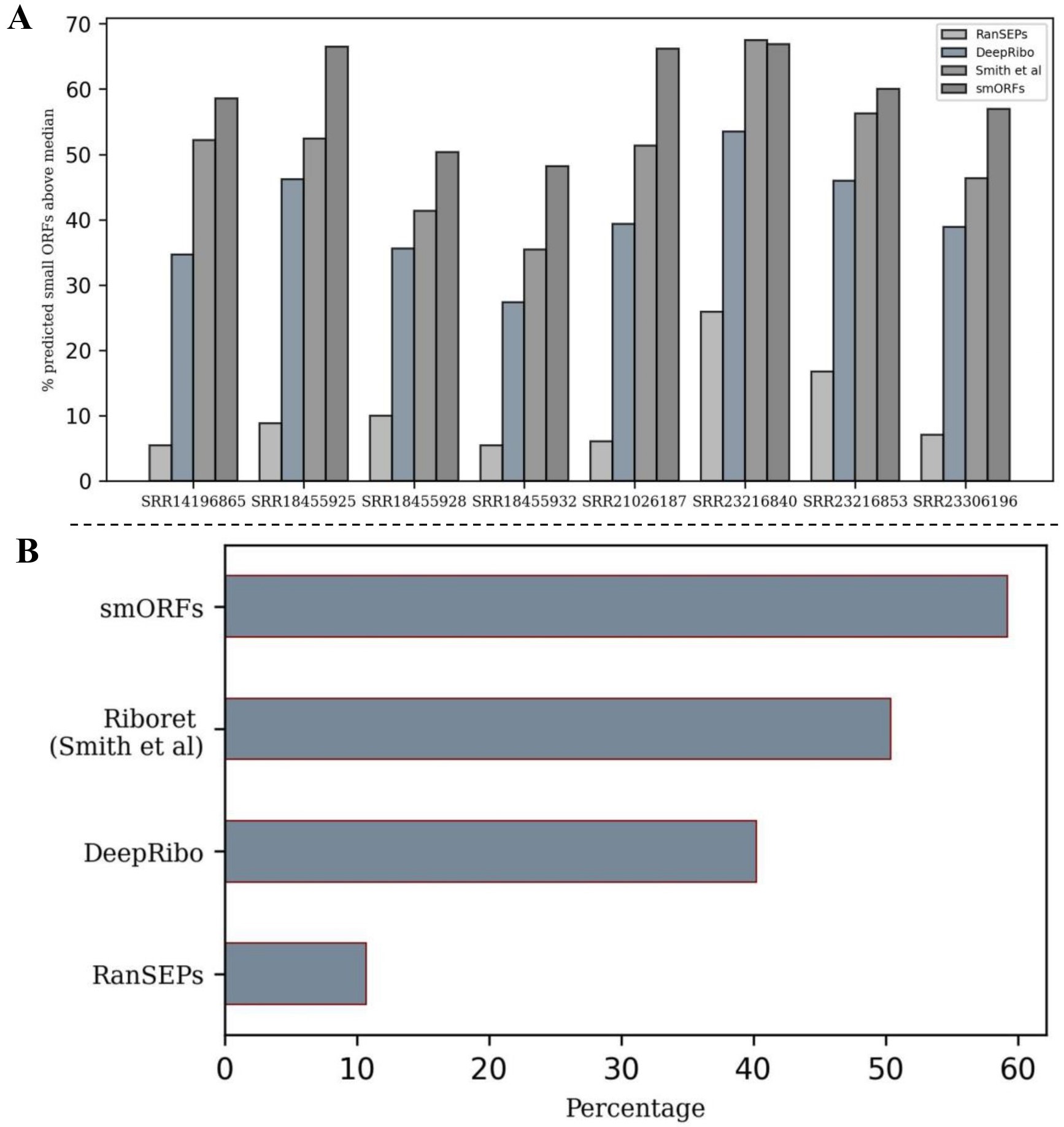

Supplement: Supplementary Figure 3 — Assessing the performance of different small protein prediction methods by evaluating ORF expression in diverse growth conditions. (A) The percentage of small proteins crossing the median expression level of annotated CDS regions in each of the eight individual RNA-Seq conditions is calculated and represented in the Y-axis, (B) The results from (A) are averaged to represent the consolidated performance for each method. [file Image_3.JPEG]

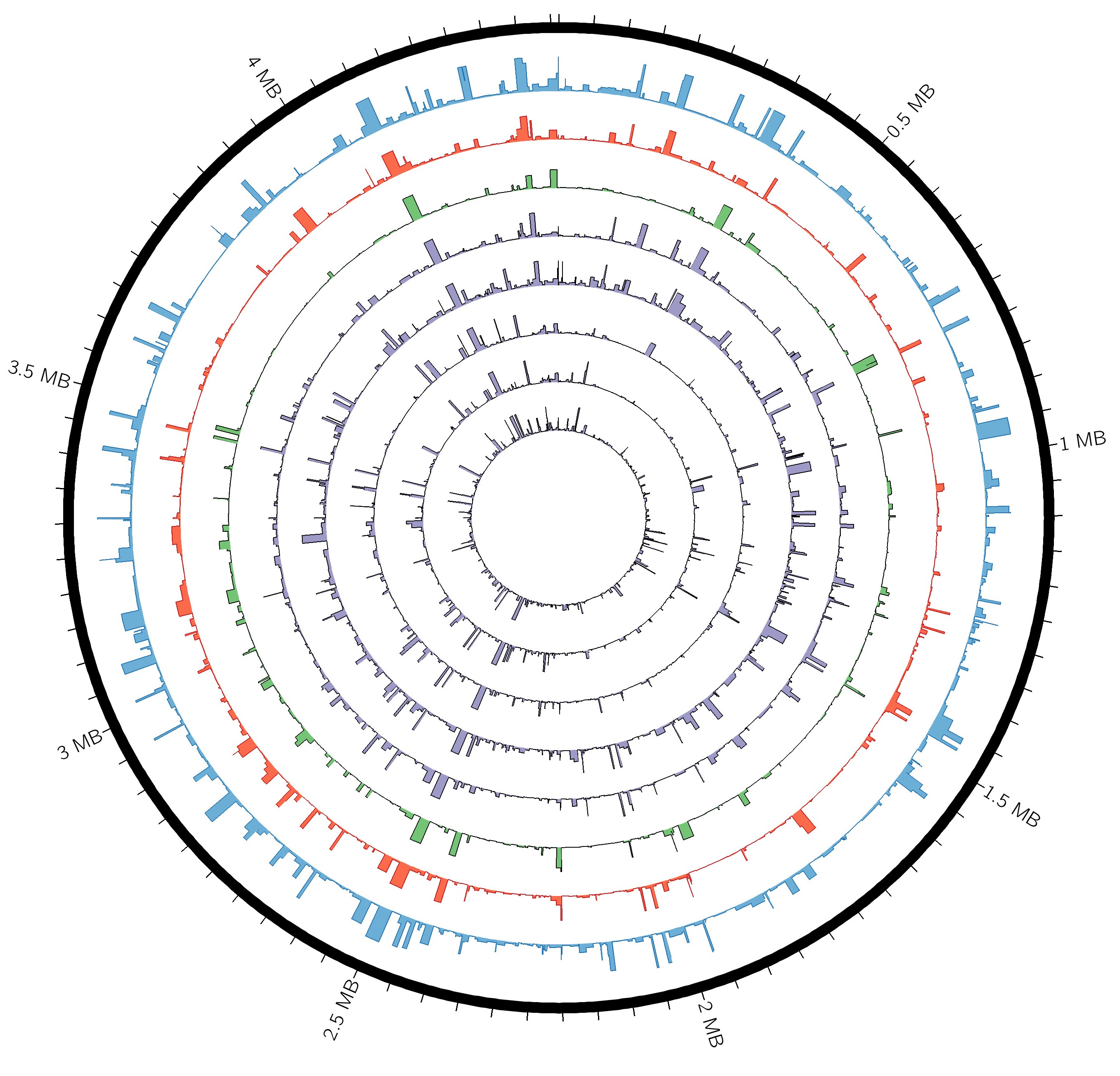

Supplement: Supplementary Figure 4 — Circular plot illustrating context-dependent expression of the smORFs in 8 distinct RNA-Seq data, ordered from the outermost to the inner circle as specified in Supplementary Table 2. The plot is generated using the Circos tool (Krzywinski et al., 2009). [file Image_4.JPEG]

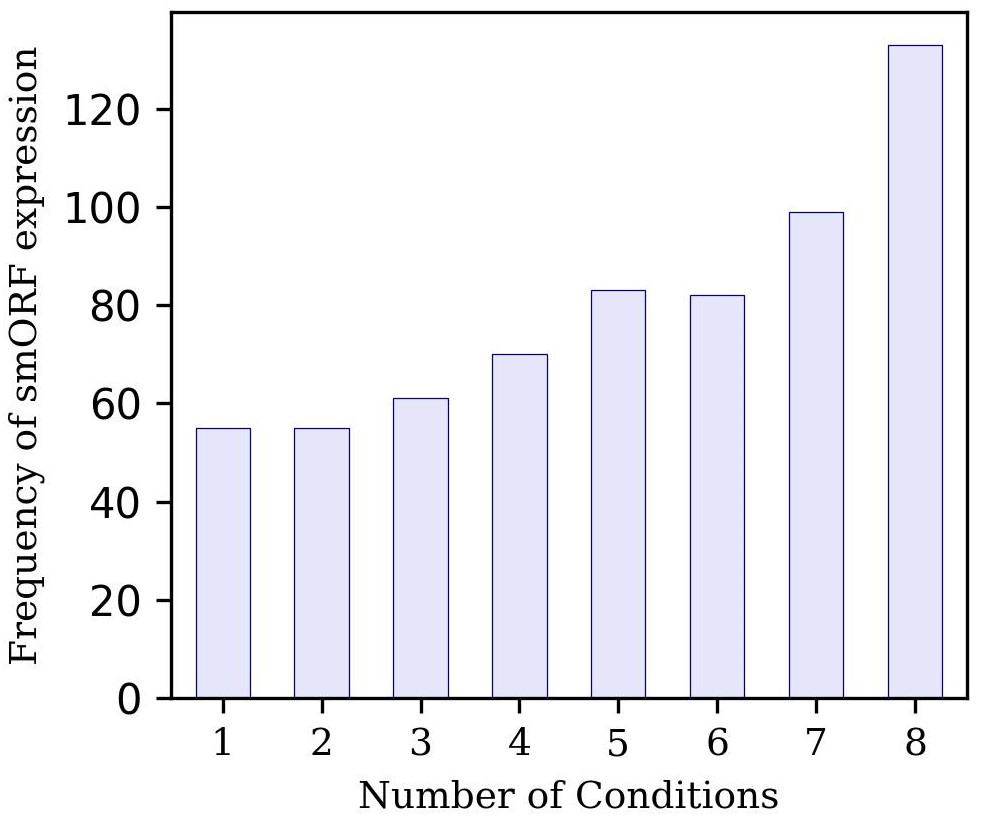

Supplement: Supplementary Figure 5 — Each bar represents the count of smORFs that are significantly expressed in a varying number of conditions. The last column indicates that 133 smORFs are significantly expressed in all eight conditions. [file Image_5.JPEG]

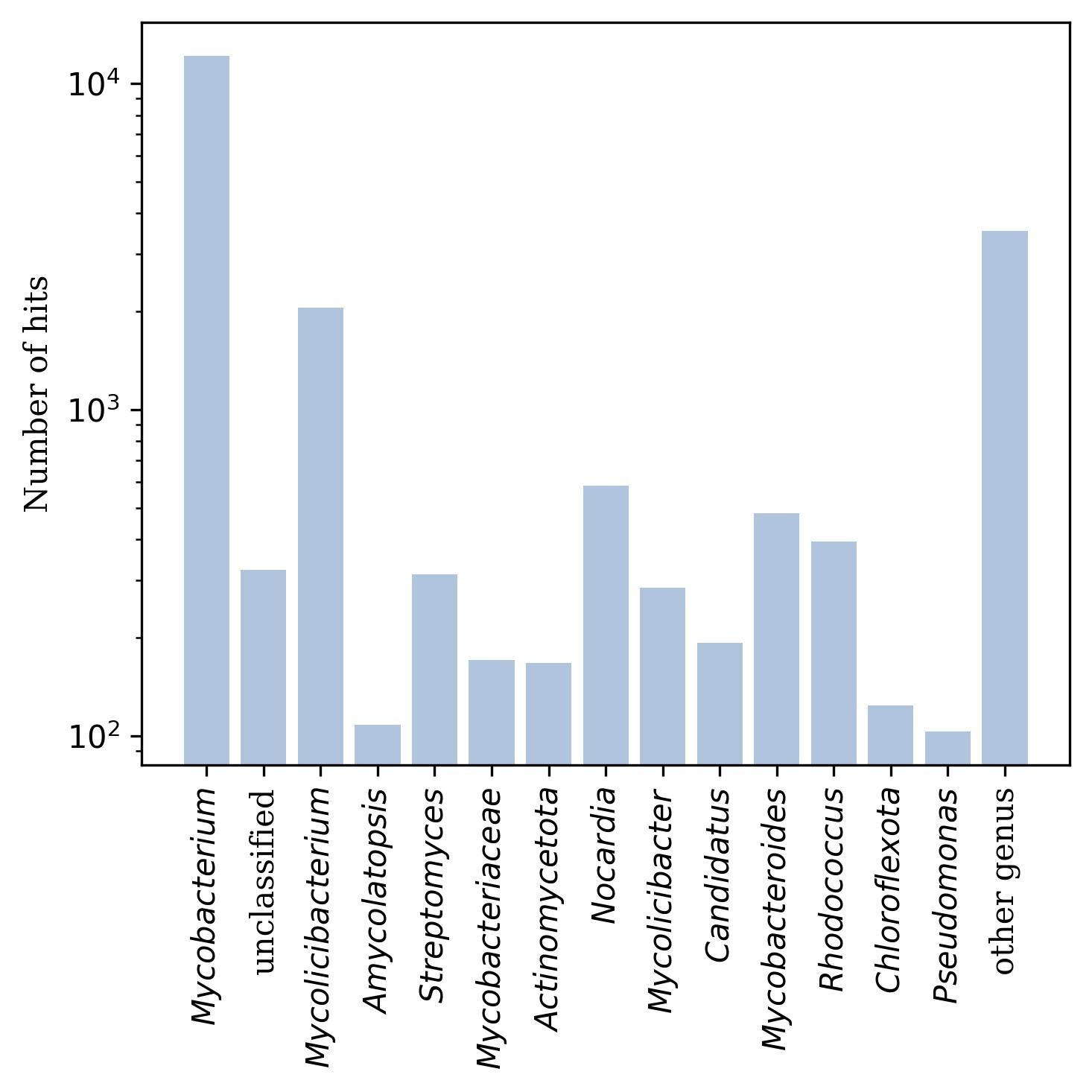

Supplement: Supplementary Figure 6 — Distribution of the hits obtained for the smORFs from Blastp against the nr database across bacterial genera. The Y-axis denotes the number of Blastp hits. [file Image_6.JPEG]

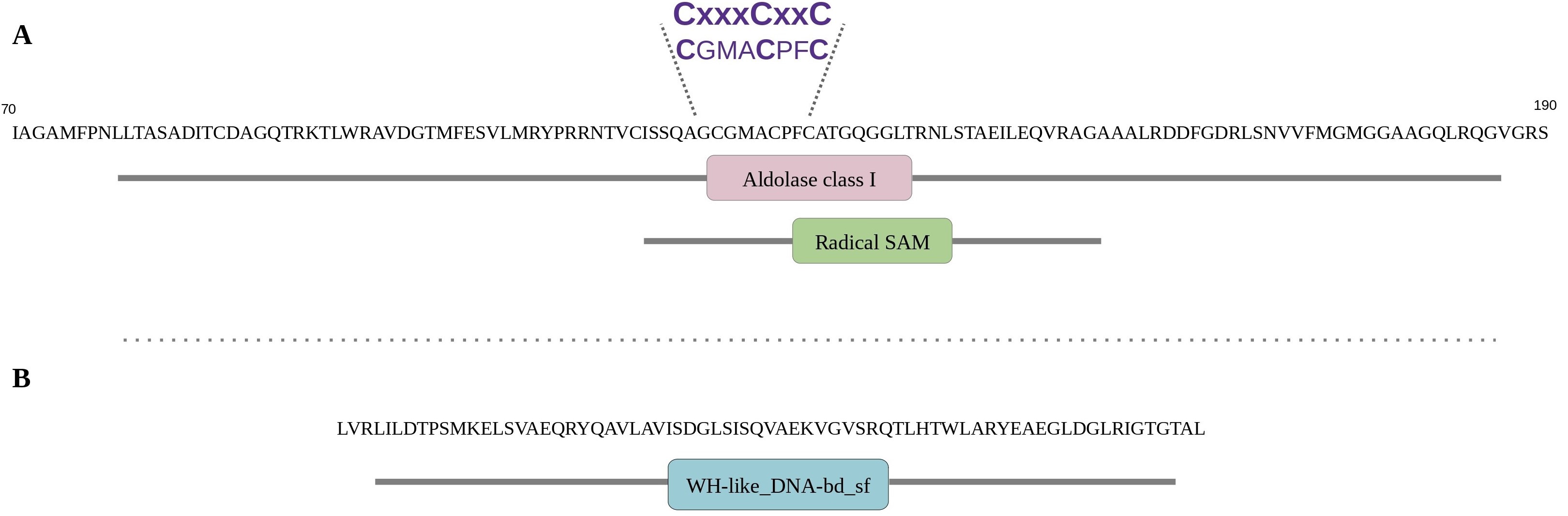

Supplement: Supplementary Figure 7 — Protein domains highlighted in the identified smORFs. (A) Presence of conserved Aldolase class I and Radical SAM domains, along with a conserved CxxxCxxC motif within MTB_ORF_47982, and (B) Winged helix-like DNA-binding domain superfamily in MTB_ORF_68067. [file Image_7.JPEG]

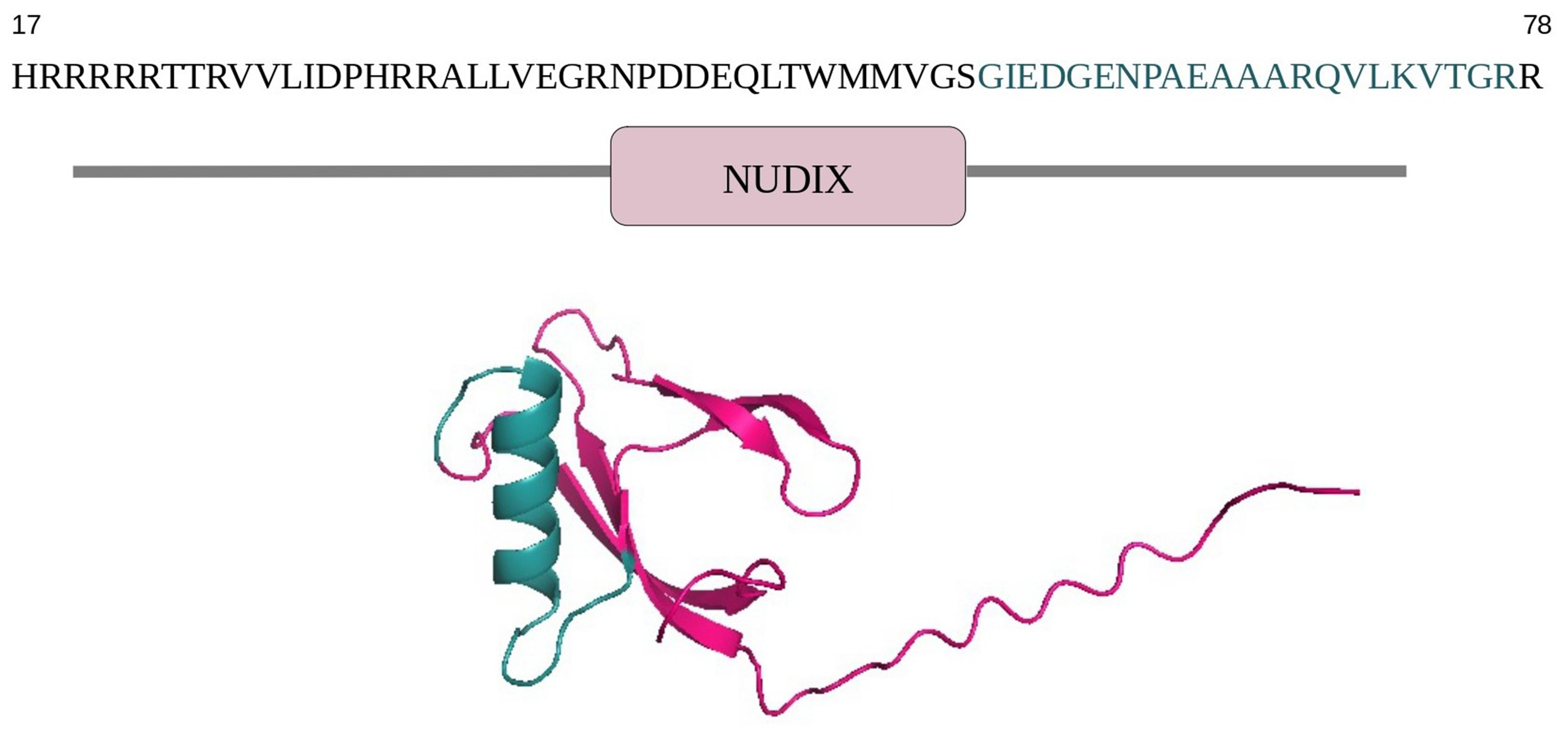

Supplement: Supplementary Figure 8 — Illustration of MTB_ORF_61001 with a NUDIX domain in protein sequence and structure (highlighted in teal). The model was built using SWISS-MODEL taking A0A7G7DNV8 (Alphafold) as a template. [file Image_8.JPEG]

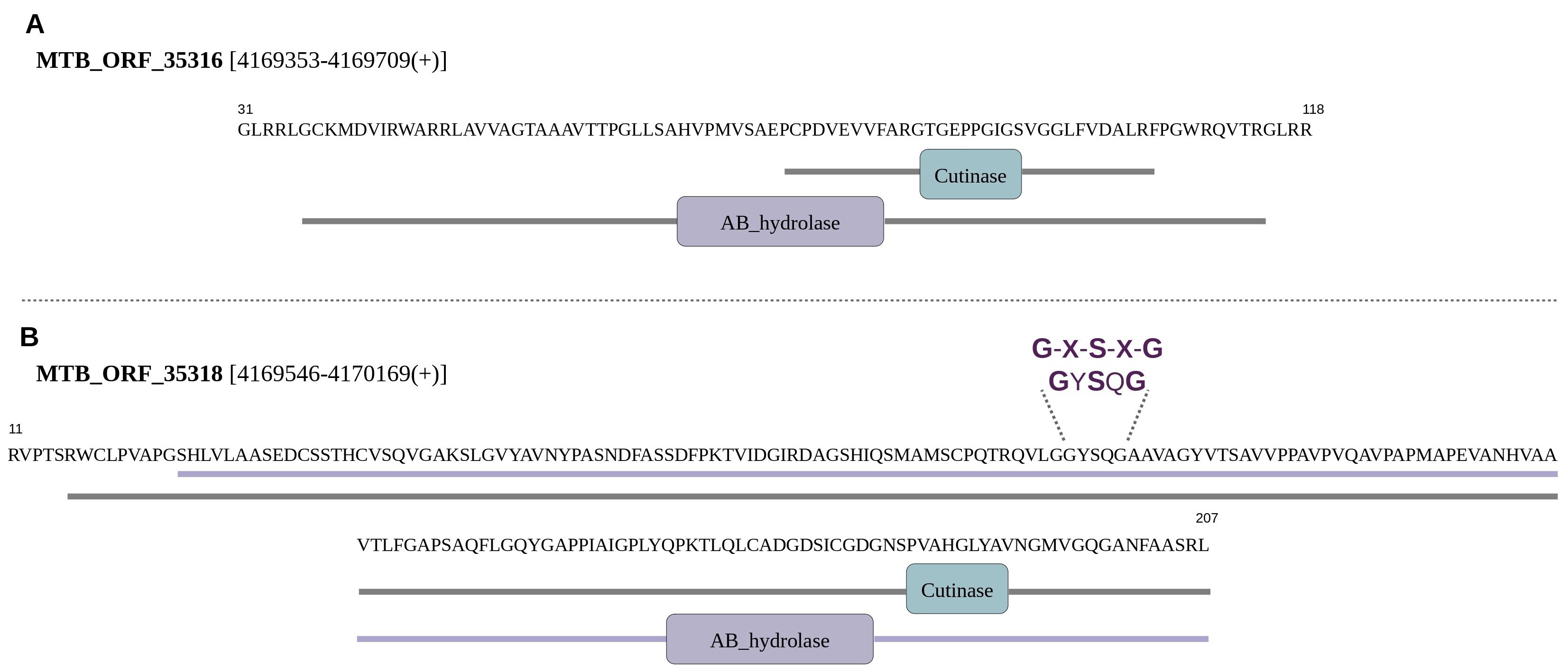

Supplement: Supplementary Figure 9 — Representation of the (A) Cutinase and AB_hydrolase domains in MTB_ORF_35316; (B) Cutinase and AB_hydrolase domains in MTB_ORF_35318, including a conserved GxSxG motif. [file Image_9.JPEG]

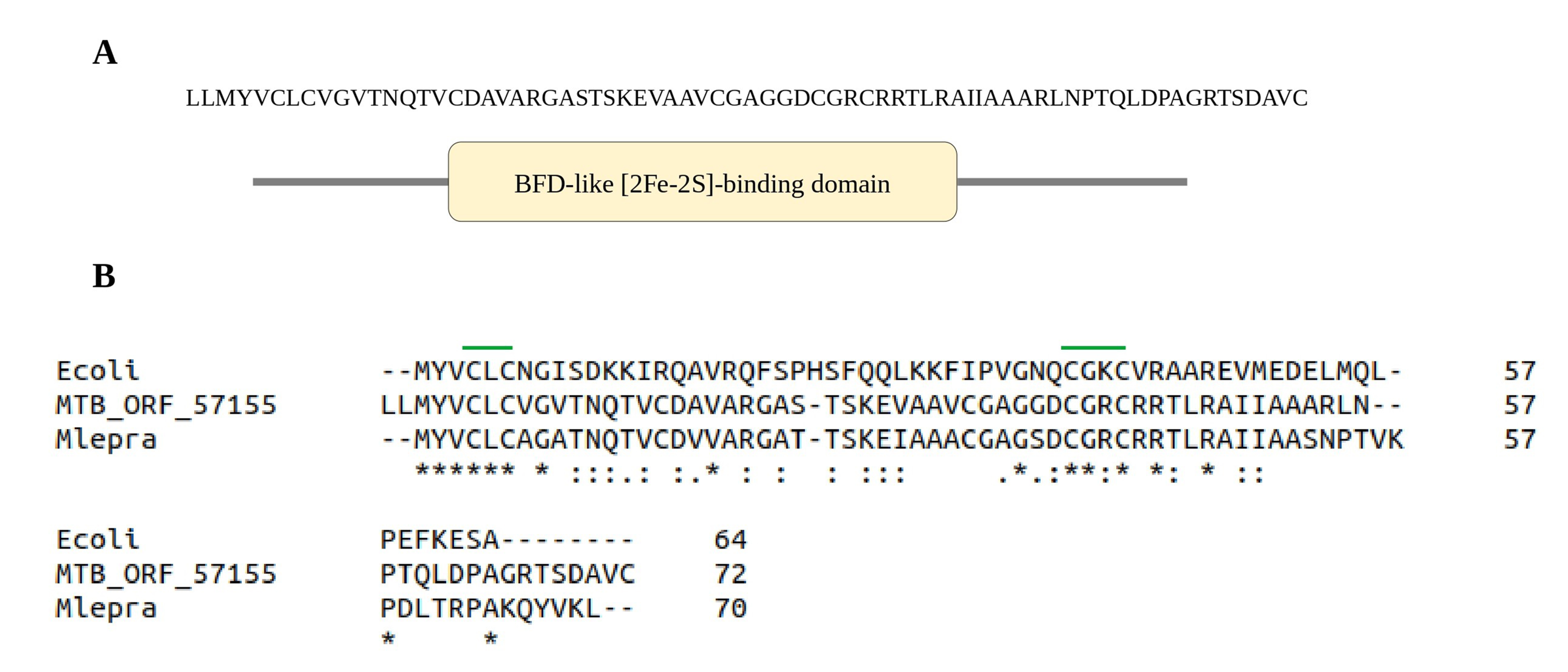

Supplement: Supplementary Figure 10 — (A) A BFD-like [2Fe-2S] domain identified within MTB_ORF_57155, and (B) Sequence alignment between BFD protein of E. coli (E.c.) (CAD6001291.1), (2Fe-2S)-binding protein of M. leprae (M.l.) (WP_081439367.1), and MTB_ORF_57155 (MORF_57155), generated using CLUSTAL O (1.2.4). The conserved cysteine residues are highlighted with green bars. [file Image_10.jpeg]

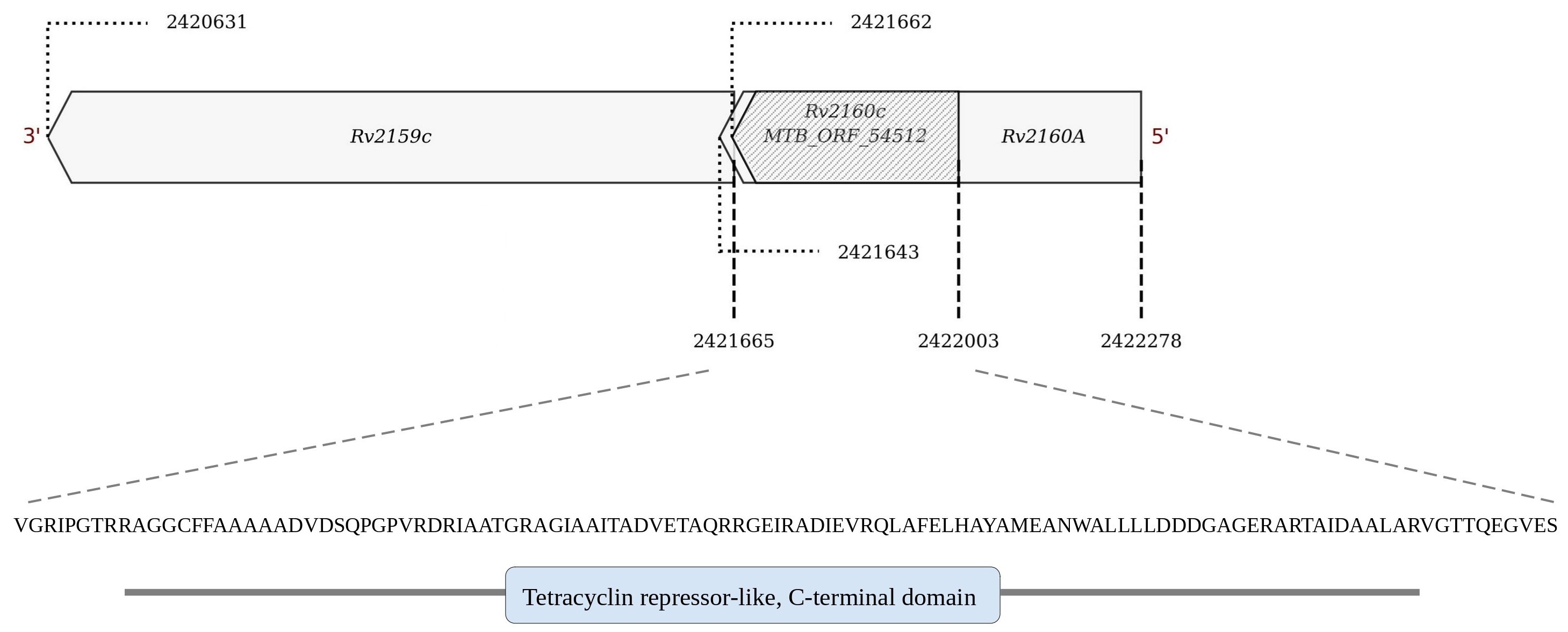

Supplement: Supplementary Figure 11 — MTB_ORF_54512 within an operon that includes Rv2159c, Rv2160c, and Rv2160A. A Tetracycline repressor-like C-terminal domain is found in MTB_ORF_54512. [file Image_11.jpeg]

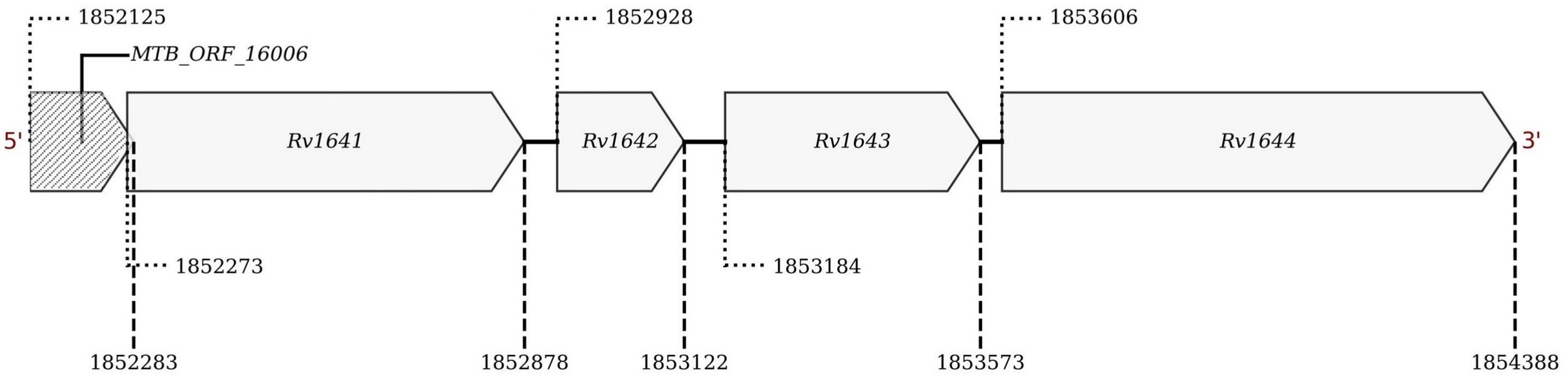

Supplement: Supplementary Figure 12 — Representation of MTB_ORF_16006 within an operon that includes the genes Rv1641, Rv1642, Rv1643, and Rv1644. [file Image_12.jpeg]
